# An Accurate Thermodynamic Model to Characterise Dissociating N_2_O_4_ at Vapour–Liquid Equilibrium States

**DOI:** 10.1007/s10765-025-03565-x

**Published:** 2025-05-10

**Authors:** Konstantin Samukov, David Vega-Maza, Eric W. Lemmon, Vladimir Diky, Silvia Lasala

**Affiliations:** 1 Université de Lorraine, CNRS, LRGP, 54000 Nancy, France; 2 Group of Energy, Economy and Systems Dynamics (GEEDS), Bioeconomy Research Institute BioEcoUVa, University of Valladolid, Paseo del Cauce 59, 47011 Valladolid, Spain; 3 Applied Chemicals and Materials Division, National Institute of Standards and Technology, Boulder, CO 80305-3337 USA

**Keywords:** Chemical equilibrium, Dinitrogen tetroxide, Equation of state, Reactive mixtures, Vapour–liquid equilibrium

## Abstract

A new thermodynamic model is presented, capable of accurately representing the vapour–liquid equilibrium pressures and densities, and liquid phase densities and enthalpies of dissociating dinitrogen tetroxide (N_2_O_4_ ⇄ 2NO_2_). The model is based on the Peng-Robinson equation of state coupled with advanced mixing rules. The -required but non-measurable- critical coordinates of the pure components forming the reactive mixtures are optimized, within a variability range defined in a previous study, to fit experimental vapour–liquid equilibrium data. The optimized parameters are then validated by comparing calculated thermodynamic properties with available experimental data in the subcritical region. The negligible impact of the higher temperature reaction 2NO_2_ ⇄ 2NO + O_2_, within the vapour–liquid equilibrium region where the optimisation is performed, is also proven. The resulting model is finally compared with the currently most accurate available equation of state, showing comparable results when considered both the scatter in available experimental data and the relative simplicity of the proposed equation of state. In particular, the proposed model demonstrates the satisfactory capability of a cubic equation of state to accurately reproduce both saturation pressures and saturation densities without requiring volume translation.

**Supplementary Information:**

The online version contains supplementary material available at 10.1007/s10765-025-03565-x.

## Introduction

When in 1957 Lighthill [1] proposed to convert the energy of dissociating gases into work, the use of N_2_O_4_ as a working fluid for power plants became a curiosity that researchers started to explore [2–7]. The N_2_O_4_ strong oxidizing capacity was also known, enabling its combination with the hydrazine family of fuels for rocket propulsion [8–10]. The benefits associated with the utilization of N_2_O_4_ in nuclear power plants were mainly investigated through research conducted at the Nuclear Power Institute of the Academy of Sciences of the Byelorussian SSR from 1960 to 1985. This Institute performed thorough studies on the reactive system N_2_O_4_ ⇄ 2NO_2_ ⇄ 2NO + O_2_, measuring its thermodynamic and transport properties in a wide range of temperature and pressure [11–13], assessing thermodynamic cycles performance theoretically and experimentally [3, 14], and investigating heat exchange processes [15, 16].

From this time to 1990, other researchers have analysed the effect of using N_2_O_4_ as a working fluid in thermodynamic cycles; a review of existing studies was conducted in Lasala et al. [17, 18]. Calculations have shown that thermodynamic cycles operating with reactive working fluids are more efficient than traditional ones based on inert fluids (H_2_O, He, and CO_2_). Furthermore, the reactive system N_2_O_4_ ⇄ 2NO_2_ has advantages in terms of heat exchange properties: comparing it with hypothetical mixtures of non-reacting N_2_O_4_ and NO_2_, it has been shown that the presence of the chemical reaction increases the heat capacity of a gas-phase mixture of N_2_O_4_ and NO_2_ up to 1000 % [19–24], the thermal conductivity up to 800 % [24–27], and the heat transfer coefficient up to 900 % [28, 29]. However, this research area died away until some researchers started revising it, proposing a multipronged approach based on fundamental thermodynamics [30], chemical reaction and plant design engineering [31], and computational fluid dynamic investigations [29]. Interest in the system N_2_O_4_ ⇄ 2NO_2_ resurfaced; however, a practical and accurate thermodynamic model of the system was still lacking.

Dinitrogen tetroxide (N_2_O_4_) rapidly dissociates into two molecules of NO_2_, or associates back to N_2_O_4_, as temperature increases or decreases, respectively, within the temperature range of approximately 260–415 K [32, 33]. The evolution of the reaction N_2_O_4_ ⇄ 2NO_2_ is dictated by the fast chemical equilibrium kinetics after a change of temperature and pressure. The dissociation of the dimer N_2_O_4_ and the association of two NO_2_ monomers are considered fast reactions: the relaxation time in a gas phase at pressures above 1 bar does not exceed 10^−4^ s [34–39]. Experimental measurements and molecular dynamics simulations conducted in the liquid phase have revealed that the reaction is likewise rapid: half-life times calculated from experimentally determined rate constants by Bauer et al. [40] fall within the range of 10^−6^ to 10^−4^ s, while molecular dynamics simulations by Katō [41] yielded a lifetime of approximately 10^−7^ s. Moreover, above about 420 K and at very low pressures for all temperatures in the vapour phase, the second reaction 2NO_2 _⇄ 2NO + O_2_ takes place and NO_2_ is totally dissociated at about 875 K [32, 33].

In the previous paper by Lasala et al. [30], we addressed the challenges of modeling fluid phase equilibrium properties of dimerization reactions of type A_n_ ⇄ (n/m)A_m_ by proposing a multi-scale methodology that was specifically applied to the binary reactive system N_2_O_4_ ⇄ 2NO_2_. This previous paper shows a characteristic feature of binary reactive mixtures: similar to pure substances, the system has a unique triple point, a unique critical point and a phase envelope where bubble and dew loci overlap to form a single “saturation” pressure–temperature curve, even though the composition changes at each point and in each phase, according to chemical equilibrium. This fact is explained by the mono-variance of the binary reactive fluid at vapour–liquid equilibrium (VLE), coincident with that of a pure fluid at saturation. In order to model the thermodynamic properties of the system with a cubic equation of state, the knowledge of critical coordinates, acentric factors and ideal gas properties of the molecules forming the mixture are required. The experimental determination of critical properties of pure N_2_O_4_ and NO_2_ is not possible because of the occurrence of the chemical reaction N_2_O_4_ ⇄ 2NO_2_ over all VLE states. To overcome this problem, we performed Monte Carlo simulations to determine the critical properties and acentric factors of the pure NO_2_ and N_2_O_4_. The ideal-gas properties of the two molecules were calculated by Quantum Mechanics simulations. Such a methodology is predictive in the sense that each input property is not optimized on experimental data but calculated by quantum and molecular approaches.

In order to increase the accuracy of the model, this paper aims, firstly, to investigate the influence of the critical properties of pure N_2_O_4_ and NO_2_, previously estimated by Lasala et al. [30], on the results of calculations of the thermodynamic properties of the reactive system. Secondly, the optimal values of these critical properties, i.e., the values that maximize the accuracy of the thermodynamic model with respect to available data, are obtained and provided. Available chemical equilibrium experimental data demonstrate that NO_2_ decomposition (2NO_2_ ⇄ 2NO + O_2_) is negligible in the subcritical domain [42–49]. That has been validated in this study, where the optimization of the model is performed within the subcritical domain, considering only the presence of the reaction N_2_O_4_ ⇄ 2NO_2_; the impact of considering the occurrence of 2NO_2_ ⇄ 2NO + O_2_ is then assessed over the subcritical and supercritical domains. After introducing in Sect. 2 the methodology that has been followed, this paper presents the results in Sect. 3.

## Methodology

The thermodynamic model and algorithms used to represent the system at chemical equilibrium are presented in Sects. 2.1 and 2.2, respectively. Section 2.3 reviews the available experimental data from the literature used in this methodology. Section 2.4 introduces the methodology for optimizing the critical coordinates of pure components to be used as an input to the thermodynamic model.

### Modelling Chemical Equilibrium for the Systems N_2_O_4_ ⇄ 2NO_2_ and N_2_O_4_ ⇄ 2NO_2_ ⇄ 2NO + O_2_

A single-reaction reactive system at chemical equilibrium is characterized by a zero-Gibbs energy of reaction, $\Delta_{R} G$, that corresponds to the minimum of the Gibbs energy of the system, $G$, with the extent of reaction variable, $\xi$, for a specific temperature, $T$, and pressure, $P$ [50, 51]:

1 $$\Delta_{R} G = \left( {\frac{\partial G}{{\partial \xi }}} \right)_{T,P} \mathop = \limits^{\begin{subarray}{l} Chem. \\ equil. \end{subarray} } 0$$

In the case where the standard state chosen for each species is the pure ideal gas at temperature T and standard pressure P°, which is the standard state that must be chosen when calculations are carried out with an equation of state, this condition translates in the following expression,

2 $$K(T)_{R} = \prod\limits_{{i = N_{comp} }} {\left( {\frac{{\hat{f}_{i} }}{{P^{ \circ } }}} \right)^{{\nu_{i} }} }$$

where $\hat{f}_{i}$ is the fugacity of component $i$, *ν*_*i*_ is the stoichiometric number of component $i$, and the equilibrium constant of the reaction R, $K(T)_{R}$, is related by its definition to the standard Gibbs energy of the considered reaction, $\Delta_{R} G^{ \circ } (T)$:

3 $$\ln K(T)_{R} = - \frac{{\Delta_{R} G^{ \circ } (T)}}{RT}$$

The condition of chemical equilibrium, Eq. 2, of the reaction N_2_O_4_ ⇄ 2NO_2_ may be rewritten as:

4 $$K(T)_{{{\text{N}}_{{2}} {\text{O}}_{{4}} \rightleftarrows {\text{2NO}}_{{2}} }} = \frac{1}{P^\circ }\prod\limits_{{i = {\text{N}}_{{2}} {\text{O}}_{{4}} {\text{,NO}}_{{2}} }} {\hat{f}_{i}^{{\nu_{i} }} = \frac{1}{P^\circ } \cdot \frac{{\hat{f}_{{{\text{NO}}_{{2}} }}^{2} }}{{\hat{f}_{{{\text{N}}_{{2}} {\text{O}}_{{4}} }} }}}$$

The condition for chemical equilibrium of the reaction 2NO_2_ ⇄ 2NO + O_2_ may be rewritten as:

5 $$K(T)_{{{\text{2NO}}_{{2}} \rightleftarrows {\text{2NO + O}}_{{2}} }} = \frac{1}{P^\circ }\prod\limits_{{i = {\text{NO}}_{{2}} {\text{,NO,O}}_{{2}} }} {\hat{f}_{i}^{{\nu_{i} }} = \frac{1}{P^\circ } \cdot \frac{{\hat{f}_{{{\text{NO}}}}^{{2}} \cdot \hat{f}_{{{\text{O}}_{{2}} }} }}{{\hat{f}_{{{\text{NO}}_{{2}} }}^{2} }}}$$

If both reactions occur simultaneously, the system at chemical equilibrium fulfils both Eqs. 4 and 5. The standard Gibbs energy of the two reactions, and thus the two equilibrium constants, are calculated from the ideal gas properties of the pure species, N_2_O_4_, NO_2_, NO and O_2_, the ideal gas standard molar enthalpy of formation at 298.15 K, the ideal gas standard molar entropy at 298.15 K, and the ideal gas isobaric heat capacity of each species [50], presented in Sect. 2.1.1. The fugacity of each species (real fluid properties) is determined by means of an equation of state presented in Sect. 2.1.2.

#### Ideal Gas Properties

A large amount of data for the ideal-gas properties of pure N_2_O_4_, NO_2_, NO and O_2_ is available in the literature. In this work, it was decided to perform chemical equilibrium calculations considering the standard molar enthalpies of formation at 298.15 K and the standard molar entropies at 298.15 K for the species N_2_O_4_, NO_2_, NO and O_2_ reported by NIST-JANAF Thermochemical Tables [52]. These properties are given in Table 1. For completeness, other available values for N_2_O_4_ and NO_2_ are provided in section S1 of the Supplementary Material.

**Table 1:** Ideal-gas properties used in this work

	N_2_O_4_	NO_2_	NO	O_2_
$\Delta_{f} H_{i,298.15\;K}^{ \circ }$/ kJ·mol^−1^	9.080	33.100	90.291	0
$S_{i,298.15\;K}^{ \circ }$/ J·mol^−1^·K^−1^	304.376	240.034	210.758	205.147

The temperature-dependent correlations for the ideal gas isobaric molar heat capacity of N_2_O_4_ and NO_2_ are taken from Lasala et al. [30], while those for NO and O_2_ are from the DIPPR database [53].

#### Equation of State

Fluid phase properties of the reactive mixture are determined with the cubic Peng-Robinson (PR) equation of state [54]:

6 $$P(T,v,{\varvec{z}}) = \frac{RT}{{v - b_{{\text{m}}} }} - \frac{{a_{{\text{m}}} }}{{v\left( {v + b_{{\text{m}}} } \right) + b_{{\text{m}}} \left( {v - b_{{\text{m}}} } \right)}}$$

where ${\varvec{z}}$ is the vector of the molar composition, $T$ is the temperature, $v$ is the molar volume, and $R$ is the universal gas constant. The mixture energy $a_{{\text{m}}}$ and co-volume $b_{{\text{m}}}$ parameters are calculated with the athermal version of the advanced EoS/$a_{res}^{E,\gamma }$ mixing rules recalled in Lasala et al. [55–57] and used in the previous paper on N_2_O_4_ from the same research group [30]:

7 $$\begin{gathered} b_{m} = \sum\limits_{i = 1}^{{N_{comp} }} {z_{i} b_{i} } \hfill \\ \frac{{a_{m} }}{{b_{m} }} = \sum\limits_{i = 1}^{{N_{comp} }} {z_{i} \frac{{a_{i} }}{{b_{i} }}} \hfill \\ \end{gathered}$$

where the pure component energy and co-volume parameters, i.e., $a_{i}$ and $b_{i}$, respectively, are calculated according to the PR equation of state as a function of the critical temperature, pressure and acentric factor of the species forming the system, as shown in Eq. 8.

8 $$\left\{ \begin{gathered} R = 8.314462618 \, J \cdot mol^{ - 1} \cdot K^{ - 1} \hfill \\ X = \left[ {1 + \sqrt[3]{4 - 2\sqrt 2 } + \sqrt[3]{4 + 2\sqrt 2 }} \right]^{ - 1} \hfill \\ b_{i} = \Omega_{b} \frac{{RT_{c,i} }}{{P_{c,i} }}{\text{ with}}\;\,\Omega_{b} = \frac{X}{X + 3} \hfill \\ a_{i} (T) = \Omega_{a} \frac{{R^{2} T_{c,i}^{2} }}{{P_{c,i} }}\left[ {1 + m_{i} \left( {1 - \sqrt {\frac{T}{{T_{c,i} }}} } \right)} \right]^{2} {\text{ with }}\left\{ \begin{gathered} \Omega_{a} = \frac{{8\left( {5X + 1} \right)}}{49 - 37X} \hfill \\ m_{i} = 0.37464 + 1.54226\omega_{i} - 0.26992\omega_{i}^{2} \hfill \\ \end{gathered} \right. \hfill \\ \end{gathered} \right.$$

For N_2_O_4_ and NO_2_, critical temperatures, pressures and acentric factors were previously calculated from the results of Monte-Carlo simulations in Lasala et al. [30], since those are not experimentally measurable, and are optimized in this work as shown in Sect. 2.4. These properties are given in Table 2, together with the measurable properties of pure NO and O_2_ taken from the DIPPR database [53].

**Table 2:** Initial critical properties and acentric factors used in this work

	$T_{c}$, K	$P_{c}$, bar	$\omega$	Reference
N_2_O_4_	484.2	55.9	0.3212	[30]
NO_2_	282.2	68.2	0.0565	[30]
NO	180.15	64.8	0.5829	[53]
O_2_	154.58	50.43	0.0222	[53]

### Algorithms Applied for Chemical Equilibrium Calculations

The knowledge of the equilibrium composition of the system is necessary to calculate the thermodynamic properties of the system in its equilibrium state. The composition can be evaluated analytically only for the simplest problems, such as ideal-gas systems [11, 58–62]. The determination of the equilibrium composition in real reactive systems, especially in the case of multiphase equilibria, requires the implementation of specific algorithms.

#### Single Phase Properties

The single-phase reactions N_2_O_4_ ⇄ 2NO_2_ and 2NO_2_ ⇄ 2NO + O_2_ or, simultaneously N_2_O_4_ ⇄ 2NO_2_ ⇄ 2NO + O_2_, take place with the number of degrees of freedom equal to two or three. The generalized Gibbs phase rule and the considered systems [63] gives:

9 $$\upsilon = 2 + c - \varphi - r - s$$

where $c$, $\varphi$ and $r$ are, respectively, the number of molecular species, the number of phases, and the number of chemical reactions. The variable $s$ is the number of additional constraints imposed on the system. The resulting variance is presented in Table 3 for the different systems of interest in the present study.

**Table 3:** Variance of the systems considered in the present study in the single phase

	N_2_O_4_ ⇄ 2NO_2_	2NO_2_ ⇄ 2NO + O_2_	N_2_O_4_ ⇄ 2NO_2_ ⇄ 2NO + O_2_
$c$	2	3	4
$\varphi$	1	1	1
$r$	1	1	2
$s$	0	0(*)	0(*)
${\varvec{\upsilon}}$	**2**	3(*)	3(*)

(*) If the molar composition of the system presents a stoichiometric relationship between NO and O_2_,

i.e. *x*(O_2_) = 2 · *x*(NO), the variable $s$ is equal to 1 and the variance ${\varvec{\upsilon}}$ is equal to 2

Calculations of chemical equilibrium correspond to the search of intensive equilibrium state variables (temperature, pressure, mole fractions) after specifying a number of independent intensive variables equal to the variance of the system. This problem has been extensively studied in the literature [64, 65]. There are two main strategies: stoichiometric and non-stoichiometric approach. In the stoichiometric approach, the condition of chemical equilibrium, Eq. 1, is considered: the Gibbs energy is minimized with respect to the extent of reaction and without additional constraints (unconstrained problem). The non-stoichiometric approach is based on direct minimization of the Gibbs energy of the system for a specified temperature, pressure and global composition with respect to material balance constraints (constrained problem). In this work, the non-stoichiometric method is applied; specifically, the modified RAND method [66, 67] is implemented to take advantage of quadratic convergence and satisfaction of the material balance constraints at every iteration.

#### Vapour–Liquid Equilibrium Properties

VLE properties of the general system N_2_O_4_ ⇄ 2NO_2_ ⇄ 2NO + O_2_ are calculated considering the presence of only the first reaction, N_2_O_4_ ⇄ 2NO_2_. It will be shown in Sect. 3.4 that the impact of the second reaction on thermodynamic properties in the VLE domain is negligible.

The reactive system N_2_O_4_ ⇄ 2NO_2_ at VLE has only one degree of freedom [30]. This fact means that only one intensive variable (temperature, pressure or the mole fraction of N_2_O_4_ or NO_2_ in either the liquid or gas phase) must be specified in order to perform equilibrium calculations. As a consequence, classical strategies of chemical equilibrium calculations can not be applied. In order to perform simultaneous phase and chemical equilibrium calculations, it is necessary to solve Eq. 10:

10 $$\left\{ {\begin{array}{*{20}c} \begin{gathered} \mu_{{N_{2} O_{4} }}^{liq} = \mu_{{N_{2} O_{4} }}^{vap} \hfill \\ \mu_{{NO_{2} }}^{liq} = \mu_{{NO_{2} }}^{vap} \hfill \\ \end{gathered} \\ {\Delta_{R} G = 0} \\ \end{array} } \right.$$

Lasala et al. [30] showed that it is enough to consider a condition of chemical equilibrium for only one of the two coexistent phases: in the case of equilibrium between phases, the condition of chemical equilibrium is automatically reached in the other phase. The implementation of an equation of state to perform calculations allows the replacement of the condition of chemical potential equality ($\mu_{i}^{{{\text{liq}}}} = \mu_{i}^{{{\text{vap}}}}$) by the condition of equal fugacity ($\widehat{f}_{i}^{liq} = \widehat{f}_{i}^{vap}$). However, after this substitution, the molar volumes of the liquid ($v^{liq}$) and vapour ($v^{{{\text{vap}}}}$) appear as two new variables of the system. Therefore, it is necessary to add in the considered system (10) two more equations connecting temperature, pressure, molar volume and composition: the equation of state applicable to both phases. As a result, we obtain a set of five equations, Eq. 11, already presented in Lasala et al. [30] and Molina et al. [68]. Indeed, the same strategy of calculations was successfully performed for the *n*-butane ⇄ isobutane isomerization by Molina et al. [68].

11 $$\left\{ {\begin{array}{*{20}c} {\hat{f}_{{N_{2} O_{4} }}^{{{\text{liq}}}} \left( {T,v^{{{\text{liq}}}} ,{\varvec{x}}} \right) = \hat{f}_{{N_{2} O_{4} }}^{{{\text{vap}}}} \left( {T,v^{{{\text{vap}}}} ,{\varvec{y}}} \right)} \\ {\hat{f}_{{NO_{2} }}^{{{\text{liq}}}} \left( {T,v^{{{\text{liq}}}} ,{\varvec{x}}} \right) = \hat{f}_{{NO_{2} }}^{{{\text{vap}}}} \left( {T,v^{vap} ,{\varvec{y}}} \right)} \\ {K(T)_{R} = \frac{1}{P^\circ }\prod\limits_{{i = N_{2} O_{4} ,NO_{2} }} {\left[ {\hat{f}_{i} \left( {T,v^{{{\text{liq}}}} ,{\varvec{x}}} \right)} \right]^{{\nu_{i} }} \;{\text{or}}\;K(T)_{R} = \frac{1}{P^\circ }\prod\limits_{{i = N_{2} O_{4} ,NO_{2} }} {\left[ {\hat{f}_{i} \left( {T,v^{{{\text{vap}}}} ,{\varvec{x}}} \right)} \right]^{{\nu_{i} }} } } } \\ {\begin{array}{*{20}c} {P - P_{{{\text{EoS}}}} \left( {T,v^{{{\text{liq}}}} ,{\varvec{x}}} \right) = 0} \\ {P - P_{{{\text{EoS}}}} \left( {T,v^{{{\text{vap}}}} ,{\varvec{y}}} \right) = 0} \\ \end{array} } \\ \end{array} } \right.$$

Vectors with molar composition of the liquid and vapour phases are denoted as ${\varvec{x}} = \left\{ {x_{{{\text{NO}}_{{2}} }} ,x_{{{\text{N}}_{{2}} {\text{O}}_{{4}} }} } \right\} = \left\{ {x_{{{\text{NO}}_{{2}} }} ,1 - x_{{{\text{NO}}_{{2}} }} } \right\}$ and ${\varvec{y}} = \left\{ {y_{{NO_{2} }} ,y_{{N_{2} O_{4} }} } \right\} = \left\{ {y_{{NO_{2} }} ,1 - y_{{NO_{2} }} } \right\}$, respectively. If all model parameters are known, system (11) has five equations and six variables: $T$, $P$,$v^{liq}$,$v^{{{\text{vap}}}}$,$x_{{{\text{NO}}_{{2}} }}$, and $y_{{{\text{NO}}_{{2}} }}$. Since the number of the degrees of freedom equals one, it is possible to fix one intensive variable and to solve the system for the five unknowns, starting from a reliable initial guess of the solution: in this work, at a specified temperature, system (11) is solved for $P$,$v^{liq}$,$v^{{{\text{vap}}}}$,$x_{{{\text{NO}}_{{2}} }}$, and $y_{{{\text{NO}}_{{2}} }}$.

In order to calculate the entire VLE curve, calculations are performed in this work from the triple point to the critical point of the system, with a specified temperature-step value. For each temperature, the system of equations is solved numerically with the Newton–Raphson method, and convergence is considered achieved for a maximum value of the residuals lower than 10^−8^; however, the implementation of this method requires suitable initial guesses. The first point of the curve is solved fixing the triple point temperature (known variable) and solving the system considering as initial guess the solution obtained analytically with the approximation «ideal gas – ideal solution»; to draw the curve at further higher temperatures, the initial guess which is used to solve the system (11) at each temperature step is the result of the previous lower temperature step calculation. Calculations are performed up to the critical point temperature, where molar compositions and molar volumes coincide with each other.

Furthermore, VLE calculations are performed assuming that N_2_O_4_ and NO_2_ do not react, in order to obtain saturated vapour pressures of pure N_2_O_4_ and pure NO_2_, and the critical line of the binary non-reactive mixture N_2_O_4_–NO_2_. The critical line of the hypothetical non-reactive system N_2_O_4_–NO_2_ is calculated with the method of Cismondi and Michelsen [69], proposed for binary non-reactive systems.

The calculated critical point of the reactive system N_2_O_4_ ⇄ 2NO_2_ is then determined as the unique point on the traced non-reactive critical line where the condition of chemical equilibrium (1) is achieved. The unique non-isothermal phase diagram of the reactive N_2_O_4_ ⇄ 2NO_2_ system at the coordinates $P - x,y$ is also drawn, and the results of calculations are compared with Monte-Carlo simulations performed in Lasala et al. [30].

### Experimental Data for the System N_2_O_4_ ⇄ 2NO_2_ ⇄ 2NO + O_2_

This section includes available experimental data for the reactive system N_2_O_4_ ⇄ 2NO_2_ ⇄ 2NO + O_2_: properties at VLE (critical properties, triple point temperature, saturated pressure, saturated densities of the liquid and vapour states, and enthalpy of vaporization). The properties of the liquid and vapour phases are presented in section S2 of the Supplementary Material.

#### Critical Properties

Experimental critical data for the considered system [70–82] are presented in Table S3 of the Supplementary Material. Critical temperature data show good agreement: experimental values from different authors differ by no more than one Kelvin, except for the data of Nadejdine [72] and Scheuer [79]. Critical pressures and critical densities are also given in Table S3, and data are in a good agreement with each other. However, the critical temperature and critical density determined by Bennewitz and Windisch [70] should be removed from consideration, the issue of this data source is addressed in Sect. 2.3.4.

#### Triple Point Temperature

The triple point temperature is necessary as a meaningful starting point for VLE pressure calculations. Experimental data on the triple point temperature are consistent with each other: Whittaker et al. [83] and Giauque and Kemp [84] reported the values 261.85 K and 261.9 K, respectively.

#### Vapour Pressure at Vapour–Liquid Equilibrium

The vapour–liquid equilibrium pressure of the considered system is very well investigated. Existing sources [13, 71, 74–79, 84–94] and the intervals of measured temperatures and VLE pressures are given in Table S4 of the Supplementary Material. 365 data points are available in the literature, 40 % of which are located in the low-temperature area between the triple point temperature and 300 K. Not all of these data are considered in the model optimization, since some sources show significant deviations from other measurements, i.e., the data of Guye and Drouginine [88] and Scheuer [79]. Indeed, VLE pressures based on the data of Guye and Drouginine [88] exhibit an inflection point near 273 K. To the knowledge of the authors, it has never been demonstrated that inflection points are excluded for reactive systems; it could be thought that at low temperatures, where the fluid is mostly made of N_2_O_4_ molecules, the saturation curve of the reactive system N_2_O_4_ ⇄ 2NO_2_ overlaps the pure N_2_O_4_ curve, while as the temperature increases and the reaction proceeds, it approaches the pure NO_2_ curve, presenting an inflection in the process. However, none of the subsequent studies have confirmed the anomalous behaviour of the curve based on data of Guye and Drouginine [88]. These data are not in agreement with the other experimental data for VLE pressure [76–78, 84, 87–90], as shown by Kulešov [95]. The calculations carried out in this study are thus conducted without the data from Scheuer [79] and Guye and Drouginine [88]. The final set of VLE pressure data considered in this work included 344 points.

#### Density at Vapour–Liquid Equilibrium

The sources and main features of the available experimental data on the saturated density of liquid and vapour phases for the reactive system N_2_O_4_ ⇄ 2NO_2_ [70, 73, 77–79, 81, 82, 89, 96–99] are presented in Tables S5 and S6 of the Supplementary Material, respectively. Tables S5 and S6 include features of the saturated densities of Reamer and Sage [77], which are not experimental data but estimates obtained from graphical processing of experimental single-phase volumetric data. Data obtained by Bennewitz and Windisch [70] and Scheuer [79], which do not agree very well with other sources, are excluded from other considerations. The final set of density data considered in this work includes 126 points for the saturated liquid phase and 85 points for the saturated vapour phase.

#### Enthalpy of Vaporization

The enthalpy of vaporization of a pure species is defined as the increase in enthalpy that accompanies the vaporization of a unit of liquid at constant temperature and pressure [100] and the calculation of this property in molar or mass terms is equivalent since the vaporization process does not change the mole number. However, this is not the case for reactive fluids in which, at constant temperature and pressure, the mole number in the liquid phase to be vaporized differs from the one of the vaporised vapour phase (see Supplementary Material, section S4). Considering that the mass of the system does not change, it is advised to present enthalpy of vaporization values on a mass basis.

In contrast to VLE pressures and densities, the enthalpy of vaporization is poorly studied for this reactive system. There are only two articles, containing overall two data: the articles of Giauque and Kemp [84] and Coon [101]. Both references report molar values, specifying the molar mass used for the conversion from mass to molar terms. With this data, it is possible to obtain the relative mass quantities: Giauque and Kemp [84] provide a value of 99 ± 0.1 cal·g^−1^ measured at 294.25 K, while Coon [101] measured a value of 73.3 cal·g^−1^ at 298.1 K.

Other sources containing data on vaporization heat [75, 76, 84, 102, 103] present results of calculations with the Clapeyron equation based on the experimental data for vapour pressure: Devojno and Mišina [102] and Seshadri et al. [103] performed calculations of the mass heat of vaporization with the experimental data for molar volumes of liquid and vapour at saturation; Scheffer and Treub [75, 76] and Giauque and Kemp [84] calculated molar heat of vaporization neglecting the volume of the liquid phase and calculating the molar volume of the vapour phase with equations of state, and evaluated the degree of dissociation from calculations based on the equilibrium constant. Data on molar vaporization heat without any additional data on chemical composition of the system should not be considered.

### Optimization of the Properties of Pure N_2_O_4_ and NO_2_

As described in the Sect. 1, in a previous work from the authors [30], coupling Quantum Mechanics and Monte Carlo simulations allowed to evaluate the thermodynamic properties of individual pure species and to perform a semi-predictive modelling of the reactive system. The term “semi-predictive” means that, although no parameters were optimized in the equation of state applied by Lasala et al. [30], Monte Carlo simulations were based on intermolecular force fields of pure NO_2_ and of pure N_2_O_4_ in turn optimized with experimental data of the reactive system by other authors [104]. Those simulations allowed the estimation of the critical coordinates and acentric factor of the pure components, with a certain degree of uncertainty [30]. To improve the accuracy of phase equilibrium properties resulting from the application of the proposed model [30], this paper proposes to optimise the critical coordinates and acentric factor of NO_2_ and of N_2_O_4_.

#### Selection of Most Sensitive EoS Input Parameters

As justified by Lasala et al. [30] and shown in Sect. 2.1.2, there are no adjustable binary interaction parameters in our model. The optimization of the model performed in this work consists in the optimal determination of the input properties required to apply the cubic equation of state: critical temperatures, critical pressures and acentric factors of NO_2_ and N_2_O_4_, keeping the ideal gas heat capacities unchanged and equal to the ones proposed by NIST [52]. It is thus possible to identify – a priori – six key parameters: critical temperatures of N_2_O_4_ and NO_2_, critical pressures of N_2_O_4_ and NO_2_, and the acentric factors of N_2_O_4_ and NO_2_.

In order to determine which of these properties should be more specifically optimized, a sensitivity analysis has initially been performed for pure-component critical temperatures, critical pressures, and acentric factors, as well as ideal-gas enthalpies and entropies of reaction at T = 298.15 K. To this aim, critical properties and acentric factors calculated in Lasala et al. [30] are initially used, with ideal gas properties provided by NIST [52]. Each parameter is perturbed by ± 10 %, keeping constant the other parameters. For each perturbed set of parameters, the system of Eq. 11 is solved to trace *T-P* and *T-ρ* diagrams. The most sensible input parameters are then chosen as parameters to be optimised, as described in Sect. 3.1.

#### Objective Function

The optimization of the parameters mentioned in Sect. 2.4.1 is performed to reproduce at best different available experimental VLE data, considering the following objective function:

12 $$F = \frac{1}{{n_{X}^{\exp } }}\sum\limits_{i = 1}^{{n_{X}^{\exp } }} {e_{X,i}^{2} } = \frac{1}{{n_{X}^{\exp } }}\sum\limits_{i = 1}^{{n_{X}^{\exp } }} {\left( {\frac{{X_{i}^{{{\text{calc}}}} - X_{i}^{\exp } }}{{X_{i}^{\exp } }}} \right)}^{2}$$

where $e_{X,i}$ is the relative deviation of the property $X$ for the *i*-th experimental point; the summation is performed over experimental data at VLE: phase boundary pressure, densities of saturated liquid and vapour, and coordinates of the critical point (critical temperature and critical pressure), where the indices “calc” and “exp” refer to calculated and experimental values, respectively. The experimental data used for the optimisation are presented in Tables S3–S6 of the Supplementary Material. Quasi-Newton BFGS (Broyden-Fletcher-Goldfarb-Shanno) is used as an optimization method. In order to verify the achievement of a global minimum, each optimization is performed 20 times for different random initializations. The success of the optimization is identified with the low number of points “out of model”, determined in accordance with available criteria [105], and low values of MAPE (Mean Absolute Percentage Error, defined as in Eq. 12). The optimisation is performed by modelling the system considering the single reaction N_2_O_4_ ⇄ 2NO_2_, while the occurrence of the second one, 2NO_2_ ⇄ 2NO + O_2_, is neglected.

#### Validation of Calculations in the Liquid Phase

Optimized parameters are then used to calculate thermodynamic properties of the system N_2_O_4_ ⇄ 2NO_2_ ⇄ 2NO + O_2_ in the liquid phase and in the supercritical region at the vicinity of the liquid phase (volumetric and caloric properties), and to validate the model’s accuracy. For specified temperatures and pressures (identical to experimental values), the molar composition $\left\{ {x_{{{\text{NO}}_{{2}} }} ,x_{{{\text{N}}_{{2}} {\text{O}}_{{4}} }} } \right\}$ is calculated by the single-phase RAND method [66, 67] and the molar volume of the liquid phase is returned by the equation of state. Calculated densities are then compared with experimental data, whose references are provided in Table S8 of the Supplementary Material. Calculation of molar concentrations $c_{i}$ are used to obtain the constants, expressed in terms of molar concentrations at equilibrium in the liquid phase $K_{{{\text{c,N}}_{{2}} {\text{O}}_{{4}} \rightleftarrows {\text{2NO}}_{{2}} }}$, according to Eq. 13, and these results are then compared with experimental data provided in Table S7 of the Supplementary Material.

13 $$K_{{c,N_{2} O_{4} \rightleftarrows 2NO_{2} }} = \frac{{c_{{{\text{NO}}_{{2}} }}^{{2}} }}{{c_{{{\text{N}}_{{2}} {\text{O}}_{{4}} }} }}$$

The enthalpy of the system N_2_O_4_ ⇄ 2NO_2_ in the liquid phase is calculated with the formula traditionally implemented for inert systems [106, 107]. The results of calculations, presented in Sect. 3, are compared with experimental data of enthalpy increment presented in Table S9 of the Supplementary Material.

## Results

This section presents the optimised input parameters and the resulting modelling capability of the equation of state, considering both the single reaction N_2_O_4_ ⇄ 2NO_2_ and the complete reactive system N_2_O_4_ ⇄ 2NO_2_ ⇄ 2NO + O_2_.

### Parametric Analysis and Parameter Optimisation

**Fig. 1:**
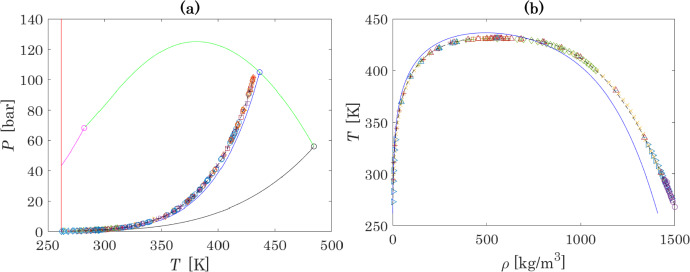
Results of calculations of VLE properties with the use of critical parameters taken from Lasala et al. [30] and ideal-gas properties from NIST-JANAF database. Figure (a) shows a *P–T* plot: pink and black curves are the vaporization curves of pure NO_2_ and N_2_O_4_, respectively; the blue curve is the calculated VLE curve of reactive N_2_O_4_ ⇄ 2NO_2_ mixture; the green curve is critical line of the binary (fictitious) inert mixture N_2_O_4_ − NO_2_; the red line corresponds to the triple point temperature; the dashed line represents the results obtained with the multiparameter equation of state introduced by Lemmon et al. [110]. Experimental data at the critical point: (✱) Schlinger and Sage [78]. Experimental data on vapour pressure at VLE: (○) Nesterenko (Ed.) [13], (◊) Greben’kov et al. [71], ( +) Scheffer and Treub [74–76], ( ×) Reamer and Sage [77], (▷) Schlinger and Sage [78], (◊) Giauque and Kemp [84], (△) Addison and Sheldon [85], (△) Baume [86], (□) Baume [87], (○) Mittasch et al. [89], ( ×) Ramsay and Young [90], ( +) Selleck et al. [91], ( −) Stoddart [92], (▷) Thorpe [93], (□) Cymarnyj [94]. Figure (b) shows a *T-ρ* plot: blue curve – calculated density curve of the reactive N_2_O_4_ ⇄ 2NO_2_ mixture; experimental data on densities coexisting at VLE: (△) Polikhronidi et al. [73], ( ×) Reamer and Sage [77], ( +) Schlinger and Sage [78], (□) Curbelev [80], ( +) Veržinskaâ et al. [82], (▷) Mittasch et al. [89], (○) Pascal [96], (◊) Greben’kov [97], ( −) Amirhanov et al. [98], (△) Veržinskaâ and Curbelev [99] (Color figure online)

The calculation of vapour pressure and densities at VLE in the N_2_O_4_ ⇄ 2NO_2_ system is firstly performed with critical properties and acentric factors previously calculated [30] from data of MC simulations performed with Brick-CFCMC software [108, 109] and ideal gas properties provided by NIST-JANAF [52]. The results are shown in Fig. 1. Input parameters are reported in Table 2. Figure 1 also presents the results obtained with the best available thermodynamic model: the Helmholtz-energy based equation of state introduced by Lemmon et al. [110], based on 111 parameters fitted to accurately represent the thermodynamic properties of the reactive system N_2_O_4_ ⇄ 2NO_2_ ⇄ 2NO + O_2_.

**Table 4:** Optimized parameters of N_2_O_4_

Parameter	Eq. constant from Lasala et al. [30]	Eq. constant from NIST-JANAF [52]
$T_{c}$(N_2_O_4_)	484.1	476.2
$P_{c}$(N_2_O_4_)	58.8	59.9

**Table 5:** MAPE of different properties in the case of non-optimised and optimised input parameters at different equilibrium constants

Property	MAPE (%) of property prediction with eq. constant from NIST-JANAF [52]		MAPE (%) of property prediction with eq. constant from Lasala et al. [30]	
	Non-optimised	**Optimised**	Non-optimised	Optimised
Equilibrium VLE pressure	18.1	**2.4**	11.9	10.7
Density of liquid at VLE	7.2	**3.5**	8.0	3.9
Density of vapor at VLE	26.3	**3.4**	9.3	9.9
Critical temperature	1.2	**0.05**	0.05	0.2
Critical pressure	3.4	**2.7**	7.2	9.3

Bold characters indicate the best optimal model

The influence of each input parameter is then investigated by comparison of the results for the initial and perturbed sets of the parameters, as described in Sect. 2.4.1. Results for each new set of parameters are shown in Figures S1–S4 of the Supplementary Material (section S3). The analysis of Figures S1–S4 shows that $T_{c}$(N_2_O_4_), $P_{c}$(N_2_O_4_)*,* $\Delta_{{\text{R}}} H_{{298.1{5}\;{\text{K}}}}^{ \circ }$ and $\Delta_{{\text{R}}} S_{{298.{15}\;{\text{K}}}}^{ \circ }$ have the highest impact on the results of calculations: parameter perturbation can cause the calculated values to change by more than 5 %.

In this work, we have thus decided to optimize only $T_{c}$ (N_2_O_4_) and $P_{c}$ (N_2_O_4_) and keep other critical properties and acentric factors equal to the values calculated with Brick-CFCMC method in Lasala et al. [30]. $\Delta_{{\text{R}}} H_{{298.1{5}\;{\text{K}}}}^{ \circ }$ and $\Delta_{{\text{R}}} S_{{298.{15}\;{\text{K}}}}^{ \circ }$ were not included in the list of optimisation parameters. However, we optimised the critical parameters for N_2_O_4_ with two sets of $\Delta_{{\text{R}}} H_{{298.1{5}\;{\text{K}}}}^{ \circ }$ and $\Delta_{{\text{R}}} S_{{298.{15}\;{\text{K}}}}^{ \circ }$ values: those derived from $\Delta_{{\text{f}}} H_{{i{,298}{\text{.15}}\;{\text{K}}}}^{ \circ }$ and $S_{{i,{298}{\text{.15}}\;{\text{K}}}}^{ \circ }$ obtained by Lasala et al. [30] and those derived from $\Delta_{{\text{f}}} H_{{i{,298}{\text{.15}}\;{\text{K}}}}^{ \circ }$ and $S_{{i,{298}{\text{.15}}\;{\text{K}}}}^{ \circ }$ published in NIST-JANAF [52].

**Fig. 2:**
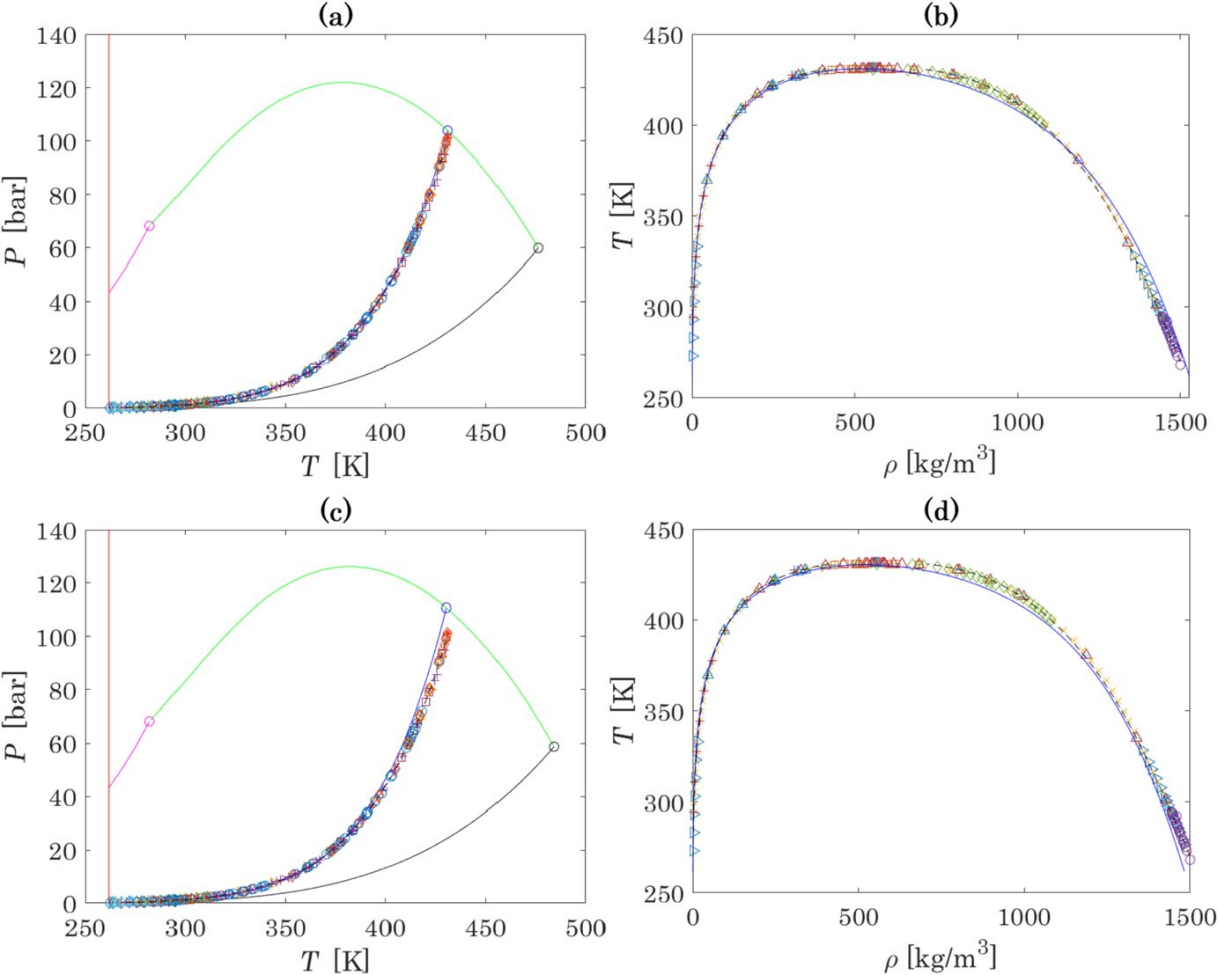
Results of the optimization of *T*_*c*_(N_2_O_4_) and *P*_*c*_(N_2_O_4_) for different equilibrium constants: Fig. (a) and (b) correspond to calculations of, respectively, the global phase equilibrium diagram and densities of the coexisting phases with the use of the expression for the equilibrium constant calculated from the NIST-JANAF database [52]. Fig. (c) and (d) correspond to the same calculations performed with the equilibrium constant obtained in Lasala et al. [30]. For more details on symbols and colours, make reference to Fig. 1

The optimal $T_{c}$ (N_2_O_4_) and $P_{c}$ (N_2_O_4_) resulting from the use of those two sets of thermochemical data are reported in Table 4. The MAPE relative to these two optimizations are presented in Table 5 and the resulting thermodynamic diagrams are presented in Fig. 2. For further comparison, Fig. 2 also shows the results obtained with the multiparameter equation of state introduced by Lemmon et al. [110].

The value of the equilibrium constant exerts a significant influence on the results of the calculations. This consideration is not surprising: the error on the equilibrium constant $K_{R}$ is exponentially related to the error on the value of $\Delta_{{\text{R}}} G^{ \circ } (T)/RT$, according to Eq. 3. The standard molar enthalpy of formation and standard molar entropy are evaluated by quantum methods and are affected by specific uncertainties that may cause important deviations between the calculated equilibrium constants [111, 112] (for example, DIPPR [53] provides an uncertainty of 3 % for heat of formation and 1 % for absolute entropy of N_2_O_4_ and NO_2_).

The analysis of Fig. 2 and Table 5 leads to the observation that the thermochemical properties that allow the best representation of experimental data (Fig. 2a and b) are the ones proposed in NIST-JANAF [52], and are thus the ones that must be used in the proposed model, together with the associated optimal critical parameters. The final set of parameters proposed in this work is given in Table 6.

**Table 6:** Final set of parameters for N_2_O_4_ and NO_2_

	N_2_O_4_	NO_2_
$T_{c}$, K	476.2 (this work)	282.2 ([30])
$P_{c}$, bar	59.9 (this work)	68.2 ([30])
$\omega$	0.3212 ([30])	0.0565 ([30])
$\Delta_{{\text{f}}} H_{{i,298.15\;{\text{K}}}}^{ \circ }$, kJ·mol^−1^	33.100 ([52])	9.080 ([52])
$S_{{i,298.15\;{\text{K}}}}^{ \circ }$, J·mol^−1^·K^−1^	240.034 ([52])	304.376 ([52])
$c_{P,i}^{ \circ } (T)$, J·kmol^−1^·K^−1^ (*T*, K)	$$\begin{gathered} 67574 + 56723 \cdot \left( {\frac{{{{723.73} \mathord{\left/ {\vphantom {{723.73} T}} \right. \kern-0pt} T}}}{{\sinh ({{723.73} \mathord{\left/ {\vphantom {{723.73} T}} \right. \kern-0pt} T})}}} \right)^{2} \hfill \\ + 13722 \cdot \left( {\frac{{{{2093.4} \mathord{\left/ {\vphantom {{2093.4} T}} \right. \kern-0pt} T}}}{{\cosh ({{2093.4} \mathord{\left/ {\vphantom {{2093.4} T}} \right. \kern-0pt} T})}}} \right)^{2} \hfill \\ \end{gathered}$$ ([30])	$$\begin{gathered} 33631 + 24566 \cdot \left( {\frac{{{{1168.4} \mathord{\left/ {\vphantom {{1168.4} T}} \right. \kern-0pt} T}}}{{\sinh ({{1168.4} \mathord{\left/ {\vphantom {{1168.4} T}} \right. \kern-0pt} T})}}} \right)^{2} \hfill \\ + 10406.5 \cdot \left( {\frac{{{{606.3} \mathord{\left/ {\vphantom {{606.3} T}} \right. \kern-0pt} T}}}{{\cosh ({{606.3} \mathord{\left/ {\vphantom {{606.3} T}} \right. \kern-0pt} T})}}} \right)^{2} \hfill \\ \end{gathered}$$ ([30])

One of the main drawbacks of cubic equations of state is their recognised incapability to describe liquid phase densities with low deviations [113]. The results obtained in this work show that the representation of this system is an exception to the rule, where saturation pressures and densities are represented with unexpected accuracy by a cubic equation of state, without the need of a translation term [114]. Results obtained for vapour pressure, density of saturated liquid and critical density in this work are significantly better than the average performance resulting from the application of an untranslated PR EoS [115].

**Fig. 3:**
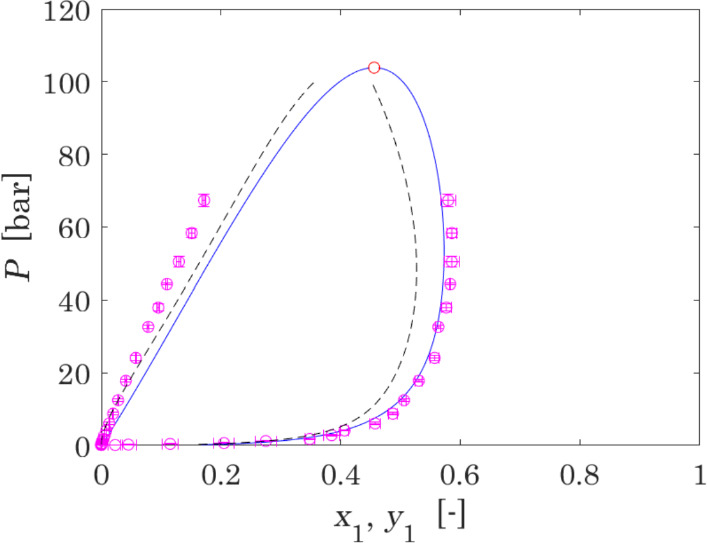
*P − *{*x*_*1*_*,y*_*1*_} diagram of the reactive mixture N_2_O_4_ ⇄ 2NO_2_, where NO_2_ is component “1”: the blue curve represents VLE calculations performed with the model proposed in this work, and the red point is the calculated critical point; pink points with error bars are the results of Monte-Carlo simulations obtained in Lasala et al. [30] by the use of Brick-CFCMC, with their uncertainties; dashed line represents the results obtained with the multiparameter equation of state introduced by Lemmon et al. [110] (Color figure online)

The resulting non-isothermal phase diagram in the coordinates *P − *{*x*_*1*_*,y*_*1*_} is shown in Fig. 3, together with Monte-Carlo simulations obtained by Lasala et al. [30], and the results of the model proposed by Lemmon et al. [110] for comparison. The prediction of the two models is not in very good agreement. However, the lack of experimental compositional data of bubble and dew points does not allow to attest which model performs best. Nevertheless, the attested deviation between the compositions predicted by the two models is expected to have a negligible impact on the size and performance assessment of the engineering processes based on dissociating N_2_O_4_.

### Validation of the Thermodynamic Model Accuracy

**Fig. 4:**
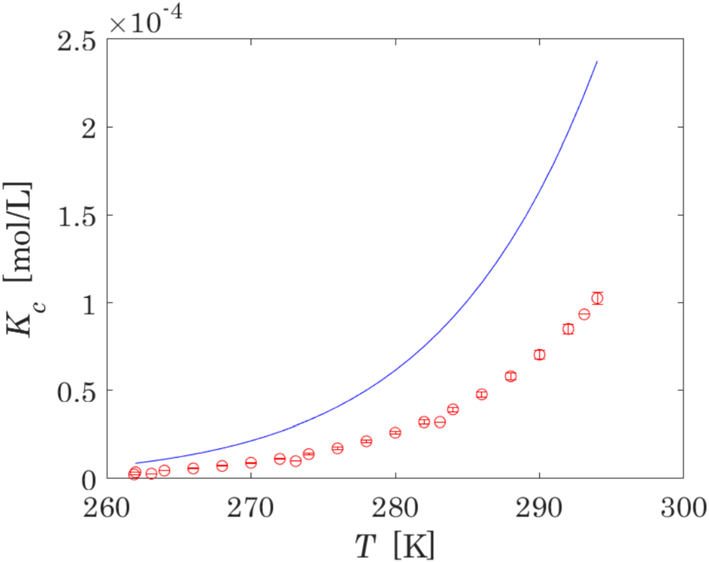
Constants in terms of concentration at equilibrium of the reaction N_2_O_4_ ⇄ 2NO_2_ in the liquid phase. Red points with error bars represent experimental data with their uncertainties [46, 49]; blue line represents calculated values (Color figure online)

The proposed optimised model has been applied to evaluate different thermodynamic properties of N_2_O_4_ ⇄ 2NO_2_ in the liquid phase. The constants calculated in terms of concentration at equilibrium at standard pressure are shown in Fig. 4.

The analysis of Fig. 4 shows that the calculated constants are not in very good agreement with experimental values. However, experimental data on chemical equilibrium are obtained under specific assumptions [61], with reported uncertainties of experimental data that are not always reliable; in addition, impurities and instrument errors could lead to inaccuracies that exceed the uncertainties provided by estimation methods [111]. The equilibrium constants obtained here proposed, in turn, may be biased to compensate for errors in other parameters and model approximations. Therefore, we can state that the optimized model provides a satisfactory description of experimental data on chemical equilibrium of the system investigated.

**Fig. 5:**
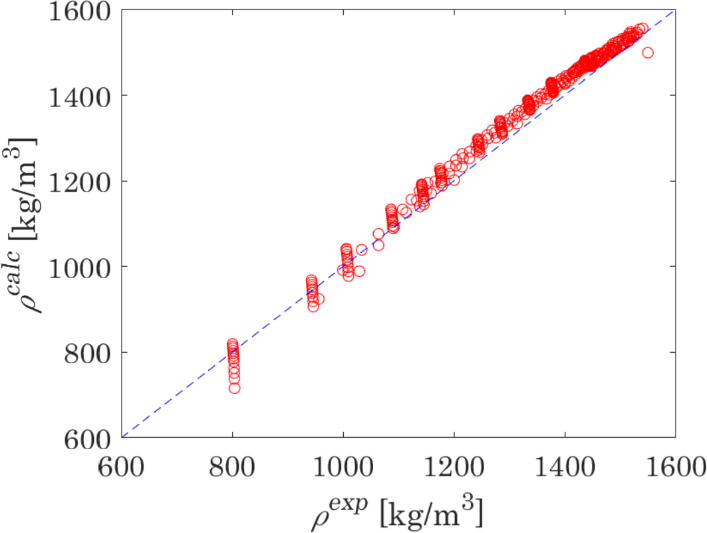
Plot comparing density calculated with the optimised model versus experimental measurements [77, 93, 94, 117–122] for the single-phase liquid in the reactive N_2_O_4_ ⇄ 2NO_2_ system

**Fig. 6:**
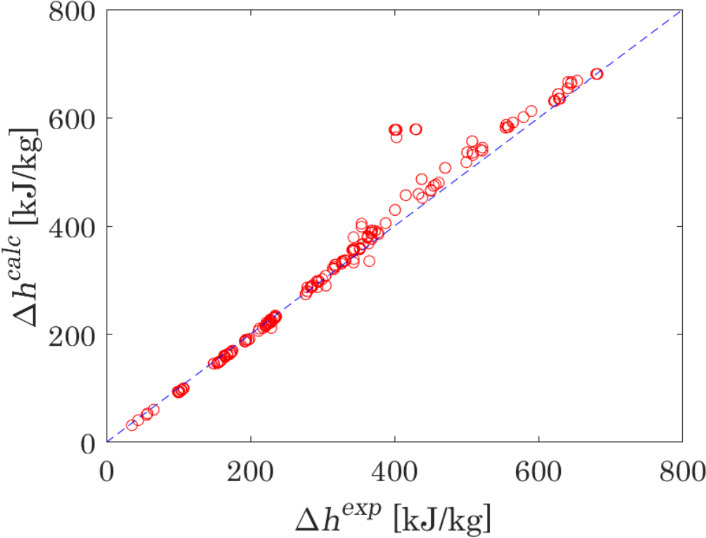
Plot comparing enthalpy change calculated with the optimised model versus experimental measurements [123–129] for the reactive N_2_O_4_ ⇄ 2NO_2_ system

Furthermore, the model is validated on liquid density and liquid enthalpy change, $\Delta h = h(T_{{{\text{out}}}} ,P) - h(T_{{{\text{in}}}} ,P)$, where $T_{{{\text{in}}}}$ and $T_{{{\text{out}}}}$ are the inlet and outlet temperatures of the calorimeter and $P$ is the pressure in the calorimeter. The results of calculations versus experimental data are presented in Figs. 5 and 6. The capability of the model to represent these properties is very satisfactory: the MAPE of liquid densities is equal to 2.8 %, which is far below deviations typically observed for non-translated PR EoS (about 7 % [116]); the MAPE of enthalpy changes is equal to 5.2 %, where several points showing the highest deviations lay in the vicinity of the calculated critical point.

The results of the calculations of thermodynamic properties in this work were compared to the results obtained with the Helmholtz energy equation of state by Lemmon et al. [110]. A comparison of the MAPEs obtained with the use of the model presented in this work and by Lemmon et al. is presented in Table 7. These MAPE are calculated considering the same experimental data, whose sources are reported in the Supplementary Material. The comparison demonstrates that, despite its simplicity, the proposed model is an effective and robust method to calculate thermodynamic properties of the considered reactive system in the subcritical region.

**Table 7:** MAPE of VLE and liquid phase properties of dissociating N_2_O_4_, calculated with two models

Property	This work	Lemmon et al. [110]
MAPE (%) of properties at VLE		
VLE pressure	2.4	0.6
Saturated liquid density	3.5	1.6
Saturated vapour density	3.4	4.5
MAPE (%) of properties in the liquid phase		
Density	2.9	0.5
Enthalpy	5.2	20.5

**Fig. 7:**
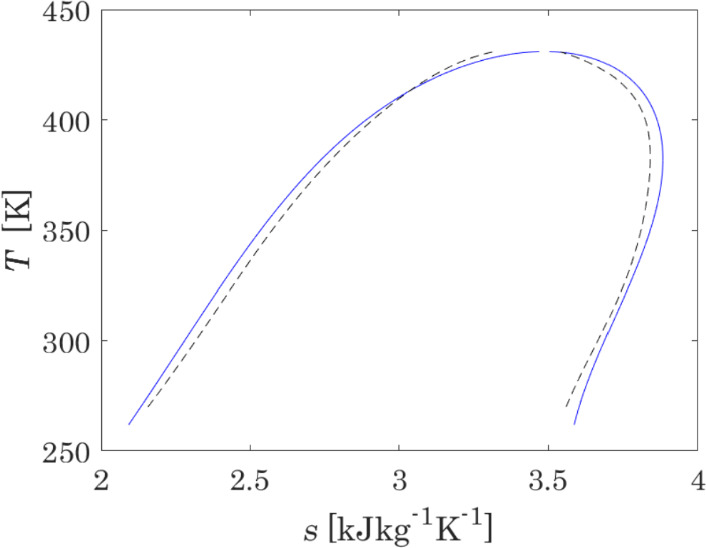
*T − s* diagram of the reactive system N_2_O_4_ ⇄ 2NO_2_: the blue solid line presents the results obtained with the model optimised in this work; the black dashed line presents the results obtained with the model by Lemmon et al. [110]

Additionally, a *T − s* diagram, also required for process simulations, is calculated and compared with the model of Lemmon et al. [110]; the results are presented in Fig. 7.

### Properties of Pure N_2_O_4_

**Fig. 8:**
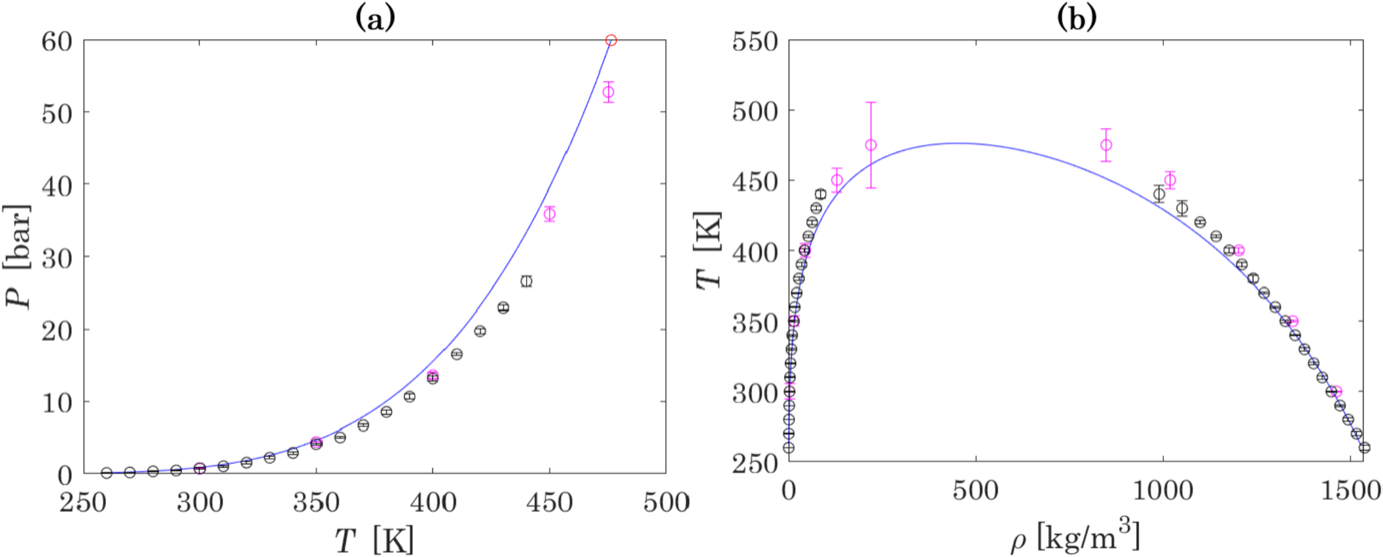
Saturated vapour pressure (a) and densities at VLE (b) of N_2_O_4_: the blue curve is based on the results of calculations with the model and parameters obtained in this work, pink points and black points present results obtained in [30] with Gibbs and Brick-CFCMC MC methods, respectively (Color figure online)

For completeness, since values of input parameters for N_2_O_4_ (critical temperature and pressure) have changed after optimization, it is worth comparing the impact of these new values on the properties of pure N_2_O_4_. Calculated values of saturated vapour pressure and density of saturated liquid of N_2_O_4_ are presented in Fig. 8. Figure 8 shows that at temperatures higher than 350 K, model predictions deviate from Monte Carlo simulation results. However, considering the uncertainty and deviations of Monte Carlo simulations themselves, the modelling result is considered satisfactory.

### Modelling the Full Reactive System N_2_O_4_ ⇄ 2NO_2_ ⇄ 2NO + O_2_

This section aims to show how the additional consideration of the dissociation of nitrogen dioxide 2NO_2_ ⇄ 2NO + O_2_ affects the results of calculations in the vapour phase. The calculations are now performed for the quaternary system N_2_O_4_ ⇄ 2NO_2_ ⇄ 2NO + O_2_ and compared to the previous case, where only N_2_O_4_ ⇄ 2NO_2_ is considered.

**Fig. 9:**
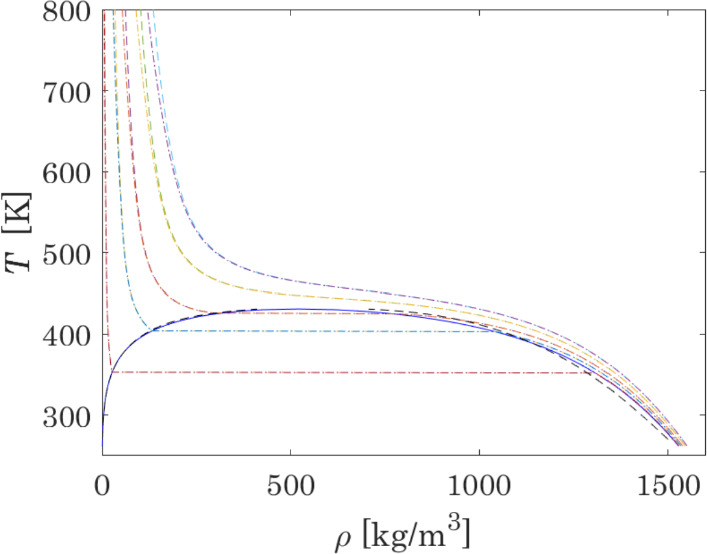
*T − ρ* diagram for a selected set of isobars (10, 50, 90, 150 and 200 bar) calculated for the system N_2_O_4_ ⇄ 2NO_2_ (dashed lines) and N_2_O_4_ ⇄ 2NO_2_ ⇄ 2NO + O_2_ (dotted lines). The blue solid line corresponds to results of VLE calculations for optimised parameters (Color figure online)

This comparison is shown on the *T − ρ* diagram in Fig. 9. It is specified that, for the quaternary system, equilibrium compositions are computed by the RAND method, as detailed in Sect. 2.2. From a volumetric point of view, it can be concluded that considering, or not, the reaction 2NO_2_ ⇄ 2NO + O_2_ does not change the results of calculations in the liquid phase and in the low-temperature vapour region. Therefore, the consideration of only the reaction N_2_O_4_ ⇄ 2NO_2_ in the subcritical region, considered in this work during the model optimisation, is proven to be a good assumption. However, at higher temperatures, where the differences between the binary or quaternary representation of the system become more pronounced, the occurrence of the second reaction should also be taken into account.

## Conclusion

This paper presents an accurate thermodynamic model based on the Peng-Robinson equation of state, coupled with the athermal version of advanced EoS/$a_{res}^{E,\gamma }$ mixing rules, and its optimal parametrisation for the accurate description of the phase equilibrium and volumetric properties of the N_2_O_4_ ⇄ 2NO_2_ system.

Reversible dimerization reactions (i.e., N_2_O_4_ ⇄ 2NO_2_) exhibit a peculiar behaviour at vapour-liquid equilibrium: the chemical reaction N_2_O_4_ ⇄ 2NO_2_ constantly evolves with the modification of temperature, and the experimental determination of the critical properties of pure N_2_O_4_ and of pure NO_2_ is thus not possible. In a previous work [30], those properties were assessed by coupling Monte-Carlo and Quantum Mechanics to characterise N_2_O_4_ and NO_2_, and it was realised that the use of a different Monte Carlo software leads to non-negligible deviations of resulting critical coordinates.

In continuity with this previous research, the present work aims to optimise those parameters in the variability range observed in Lasala et al. [30] to improve the model’s accuracy in the two-phase region. Prior to parameter optimisation, a sensitivity analysis is performed, leading to two main conclusions: (1) the variation of thermochemical ideal gas properties, such as standard molar enthalpy of formation and standard molar entropy, in the uncertainty range characterising the most accurate Quantum Chemistry results, leads to significant deviations of thermodynamic calculations; (2) only the critical properties of N_2_O_4_ have a non-negligible impact on the calculated VLE properties of reactive mixtures. In this work, the ideal gas thermochemical properties are thus set to the values proposed by NIST [52, 130], the critical coordinates of NO_2_ are fixed equal to the values obtained in Lasala et al. [30], while the critical coordinates of the N_2_O_4_ are optimised.

The model’s optimisation is performed by considering the occurrence of the only reaction N_2_O_4_ ⇄ 2NO_2_. Furthermore, the validity of such an assumption is confirmed by modelling the full reactive system N_2_O_4_ ⇄ 2NO_2_ ⇄ 2NO + O_2_ at VLE and attesting the negligible variation of the calculations due to the inclusion of the higher-temperature reaction 2NO_2_ ⇄ 2NO + O_2_. Moreover, the optimised model is finally validated based on liquid densities and enthalpy variations, and compared with the results obtained with the Helmholtz energy equation of state recently proposed by Lemmon et al. [110].

Despite its simplicity, the here-parametrised cubic equation of state enables the description of VLE densities and pressures, with an excellent degree of accuracy, which is quantified in this paper. The obtained equation of state thus enables the reliable design and optimisation of chemical, power generation and refrigeration processes based on N_2_O_4_ ⇄ 2NO_2_.

## Supplementary Information

Below is the link to the electronic supplementary material.

Supplementary file1 (DOCX 541 KB)

## Data Availability

Data will be made available upon request.
