# Supplementary material for: An Accurate Thermodynamic Model to Characterise Dissociating N2O4 at Vapour–Liquid Equilibrium States
Source: Int J Thermophys. 2025 May 10;46(7):95. doi: 10.1007/s10765-025-03565-x (PMC12065768; doi:10.1007/s10765-025-03565-x)
Supplement: Supplementary file 1 — Supplementary file1 (DOCX 541 KB) [file 10765_2025_3565_MOESM1_ESM.docx]

**Supplementary Material of the paper:**

**An accurate thermodynamic model to characterise dissociating N_2_O_4_ at vapour-liquid equilibrium states**

Konstantin Samukov^†^, David Vega-Maza^‡^, Eric W. Lemmon^§^,[[1]](#footnote-1), Vladimir Diky^§,1^

and Silvia Lasala^†,*^

† *Université de Lorraine, CNRS, LRGP, F-54000 Nancy, France.*

‡ *Group of Energy, Economy and Systems Dynamics (GEEDS), Bioeconomy Research Institute BioEcoUVa, University of Valladolid, Paseo del Cauce 59, 47011 Valladolid, Spain*.

§ *Applied Chemicals and Materials Division, National Institute of Standards and Technology, Boulder, Colorado 80305-3337, USA.*

** E-mail:* [*silvia.lasala@univ-lorraine.fr*](mailto:silvia.lasala@univ-lorraine.fr)

**S1. Ideal gas properties**

**Table S1.** Available experimental data on standard molar enthalpy of formation and standard molar entropy for pure N_2_O_4_ and NO_2_ at 298.15 K.

| Source | $\Delta_{f}H_{298.15 K}^{\circ}$, kJ/mol | | $S_{298.15 K}^{\circ}$, J/mol/K | |
| --- | --- | --- | --- | --- |
|  | N_2_O_4_ | NO_2_ | N_2_O_4_ | NO_2_ |
| Chao [1] | 9.16 | 33.18 | 304.64 | 240.25 |
| Hisatsune [2] | 10.63 | − | 304.30 | − |
| Altshuller [3] | − | − | − | 239.83 |
| Gordon [4] | − | − | − | 239.94 |
| Lasala et al. [5] | 9.99 | 34.20 | 297.65 | 240.03 |
| Gurvich et al. [6] | 11.11 | 34.19 | 304.35 | 240.06 |
| NIST-JANAF [7] | 9.080 | 33.100 | 304.376 | 240.034 |

**Table S2.** Available experimental data on standard molar enthalpy and standard molar entropy for the reaction N_2_O_4_ ⇄ 2NO_2_ at 298.15 K.

| Source | $\Delta_{R}H_{298.15 K}^{\circ}$, kJ/mol | $\Delta_{R}S_{298.15 K}^{\circ}$, J/mol/K |
| --- | --- | --- |
| Chao [1] | 57.20 | 175.86 |
| Hisatsune [2] | 57.07 | 175.35 |
| Lasala et al. [5] | 58.41 | 182.41 |
| Gurviĉ et al. [6] | 57.27 | 175.71 |
| NIST-JANAF [7] | 57.12 | 175.68 |
| Blend [8] | 57.11 | − |
| Harris and Churney [9] | 57.07 | − |
| Mišina et al. [10] | 57.57 | − |
| Vosper [11] | 57.11 | 175.39 |
| Glendening and Halpern [12] | 58.40 | 176.45 |

**S2. Available experimental data for the reactive system N_2_O_4_ ⇄ 2NO_2_ ⇄ 2NO+O_2_**

**Table S3**. Available experimental critical properties of the reactive system N_2_O_4_ ⇄ 2NO_2_ ⇄ 2NO+O_2_.

| Source | $T_{c}$, K | $P_{c}$, bar | $\rho_{c}$, kg/m^3^ |
| --- | --- | --- | --- |
| Bennewitz and Windisch [13] | 431.34 ± 0.7 | − | 570 ± 18 |
| Greben’kov et al. [14] | 430.91 | 101.4 | − |
| Nadejdine [15] | 444.34 | − | − |
| Polikhronidi et al. [16] | 431.07 ± 0.1 | − | 551.7 ± 4.33 |
| Scheffer and Treub [17–19] | 431.35 ± 0.2 | 101.33 | − |
| Reamer and Sage [20] / Schlinger and Sage [21] | 431.36 | 101.28 | 550.5 |
| Scheuer [22] | 430.1 | − | 566.7 |
| Curbelev [23,24] | 430.91 ± 0.61 | − | 556.2 |
| Veržinskaâ et al. [25] | 430.91 ± 0.1 | − | 555 ± 6 |

**Table S4**. Available P-T experimental data of the reactive system N_2_O_4_ ⇄ 2NO_2_ ⇄ 2NO+O_2_ at vapour-liquid equilibrium conditions. Data indicated with (*) have not been used as justified in the main text.

| Source | Number of points | Temperature range, K | Pressure range, bar |
| --- | --- | --- | --- |
| Greben’kov et al. [14] | 42 | 352-431 | 10-101 |
| Scheffer and Treub [17–19] | 72 | 262-431 | 0.2-1 |
| Reamer and Sage [20] | 25 | 294-428 | 1-92 |
| Schlinger and Sage [21] | 26 | 294-428 | 1-92 |
| Scheuer [22] | 13(*) | 263-288 | 0.2-0.8 |
| Nesterenko [26] | 53 | 292-427 | 1-91 |
| Giauque and Kemp [27] | 24 | 262-295 | 0.1-1 |
| Addison and Sheldon [28] | 6 | 263-293 | 0.2-1 |
| Baume and Robert [29,30] | 25 | 265-311 | 0.2-2.2 |
| Guye and Drouginine [31] | 8(*) | 263-285 | 0.2-0.8 |
| Mittasch et al. [32] | 34 | 276-333 | 0.4-5.2 |
| Ramsay and Young [33] | 17 | 263-295 | 0.2-1 |
| Selleck et al. [34] | 1 | 278 | 0.45 |
| Stoddart [35] | 8 | 273-293 | 0.3-1 |
| Thorpe [36] | 1 | 295 | 1 |
| Cymarnyj [37] | 9 | 363-428 | 14-94 |

**Table S5**. Features of available saturated liquid density data for the reactive system N_2_O_4_ ⇄ 2NO_2_ ⇄ 2NO+O_2_. Data indicated with (*) have not been used as justified in the main text.

| Source | Number of points | Temperature range, K | Density range, kg/m^3^ |
| --- | --- | --- | --- |
| Bennewitz and Windisch [13] | 19(*) | 293-431 | 738-1447 |
| Polikhronidi et al. [16] | 15 | 301-431 | 562-1427 |
| Reamer and Sage [20] | 26 | 294-431 | 550-1439 |
| Scheuer [22] | 15(*) | 283-429 | 730-1468 |
| Curbelev [24] | 4 | 430-431 | 538-682 |
| Veržinskaâ et al. [25] | 7 | 406-431 | 589-1033 |
| Mittasch et al. [32] | 28 | 276-329 | 1356-1484 |
| Pascal and Garnier [38] | 19 | 268-294 | 1444-1500 |
| Greben’kov et al. [39] | 23 | 400-431 | 556-1080 |
| Amirhanov [40] | 5 | 301-427 | 804-1427 |

**Table S6**. Available saturated vapour density data of the reactive system N_2_O_4_ ⇄ 2NO_2_. Data indicated with (*) have not been used as justified in the main text.

| Source | Number of points | Temperature range, K | Density range, kg/m^3^ |
| --- | --- | --- | --- |
| Bennewitz and Windisch [13] | 19(*) | 293-431 | 3.4-488 |
| Polikhronidi et al. [16] | 11 | 417-431 | 201-550 |
| Reamer and Sage [20] | 26 | 294-431 | 3-550 |
| Schlinger and Sage [21] | 9 | 294-428 | 3.5-306 |
| Scheuer [22] | 15(*) | 283-429 | 3.4-405 |
| Curbelev [24] | 6 | 430-431 | 429-519 |
| Veržinskaâ et al. [25] | 19 | 370-431 | 46-556 |
| Mittasch et al. [32] | 7 | 273-333 | 1.4-15.2 |
| Veržinskaâ and Curbelev [41] | 9 | 370-431 | 45-556 |

**Table S7**. Used in this work experimental data on equilibrium constants in the liquid phase of the reactive system N_2_O_4_ ⇄ 2NO_2_.

| Source | Number of points | Temperature range, K |
| --- | --- | --- |
| Gray and Rathbone [42] | 6 | 262-294 |
| James and Marshall [43] | 4 | 266-296 |
| Vosper [44] | 16 | 263-333 |

**Table S8**. Used in this work experimental data on liquid phase density of the reactive system N_2_O_4_ ⇄ 2NO_2_.

| Source | Number of points | Temperature range, K | Pressure range, bar | Density range, kg/m^3^ |
| --- | --- | --- | --- | --- |
| Reamer and Sage [20] | 98 | 294-428 | 34-414 | 956-1487 |
| Thorpe [36] | 1 | 273 | 1 | 1490 |
| Cymarnyj [37] | 117 | 299-511 | 26-594 | 800-1441 |
| Addison and Smith [45] | 23 | 263-293 | 1 | 1447-1515 |
| Ramsay and Shields [46] | 2 | 275-293 | 1 | 1444-1487 |
| Bousfield [47] | 20 | 277-293 | 1 | 1450-1484 |
| Vitûk [48] / Vitûk and Golovskij [49] / Vitûk et al. [50] | 52 | 262-343 | 23-596 | 1402-1540 |

**Table S9**. Used in this work experimental data on enthalpy increment of the liquid phase of the reactive system N_2_O_4_ ⇄ 2NO_2_.

| Source | Number of points | Temperature range, K | Pressure range, bar | Enthalpy increment range, kJ/kg |
| --- | --- | --- | --- | --- |
| Sarumov [51] | 38 | 283-429 | 49-294 | 101-402 |
| Šejndlin et al. [52,53] | 45 | 283-438 | 49-294 | 100-430 |
| Šejndlin et al. [54] | 41 | 283-431 | 49-294 | 100-430 |
| Šejndlin et al. [55] | 20 | 283-468 | 49-294 | 35-522 |
| Šejndlin et al. [56] / Simonov [57] | 69 | 283-482 | 118-294 | 44-654 |

**S3. Sensibility analysis plots**

This section introduces the results of the sensibility analysis, applied to the variables of the optimisation problem and presented in Figures S1 – S4. Pink solid curves represent results of calculations in the case of non-perturbed input parameters (thus coincident with calculations shown in Figure 1 of the main manuscript); green dashed and black dashed curves portray the results of calculations at perturbed values of the variable parameter: values of the parameter were increased or reduced by 10%, respectively. Blue curves represent the results of calculations in the case of coincidence between the results of calculations for non-perturbed and perturbed values of parameters. The red line corresponds to the triple temperature point of the reactive system.


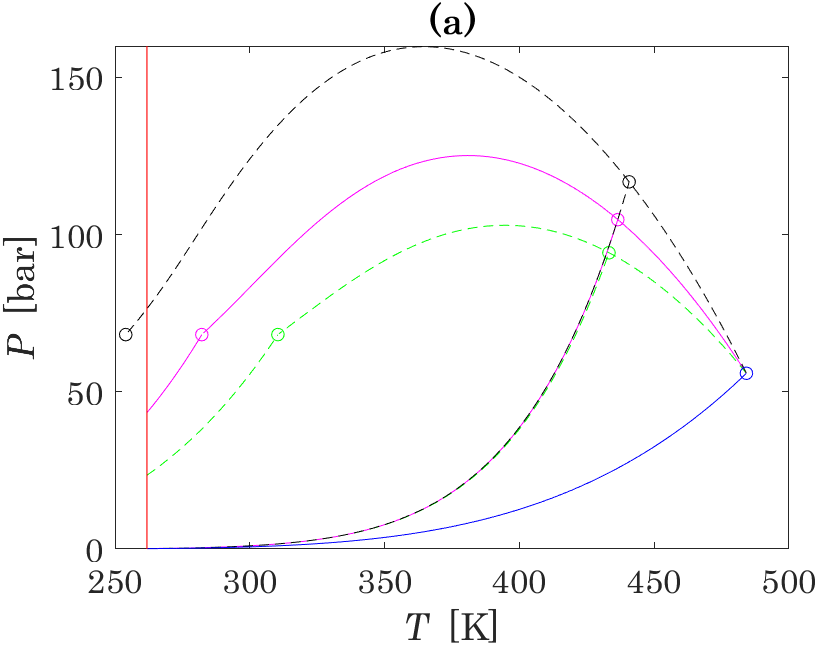

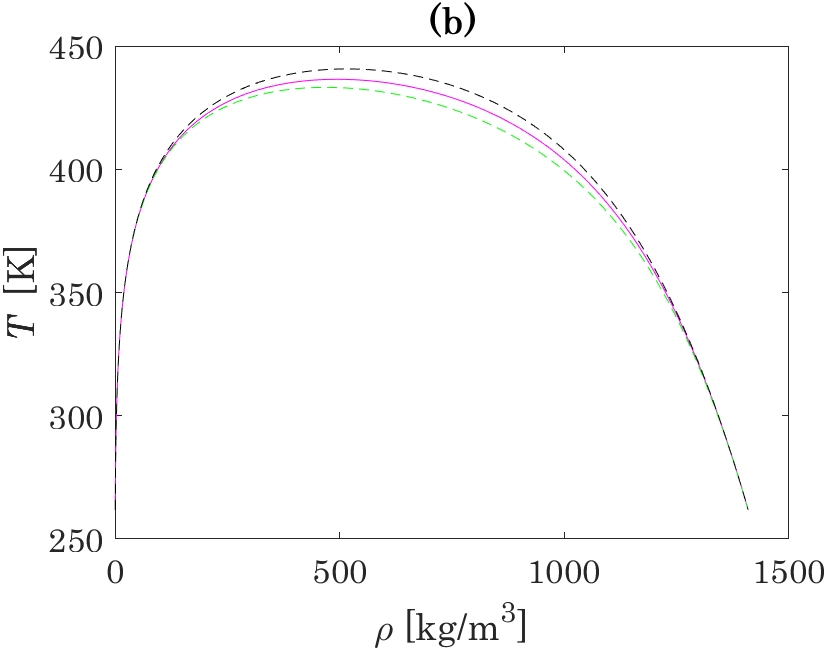


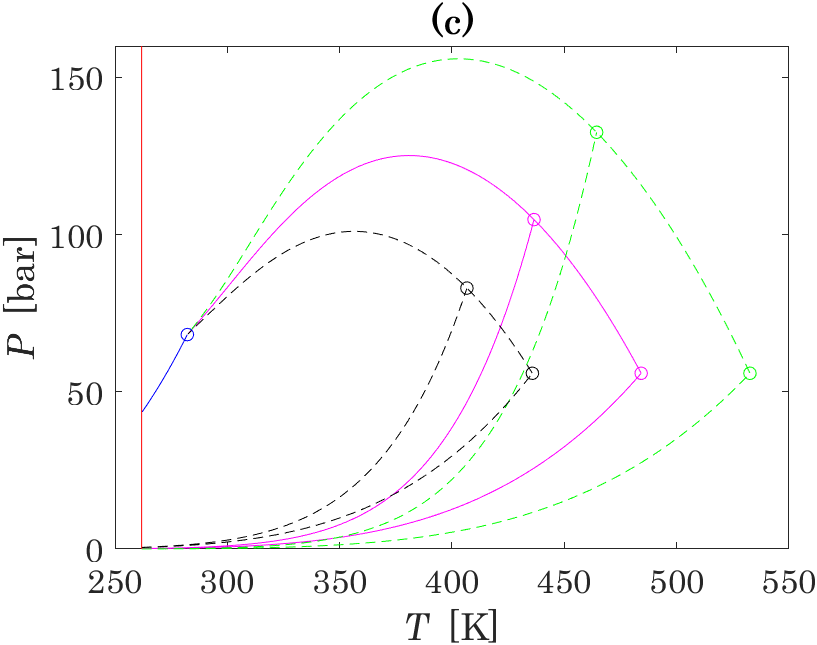

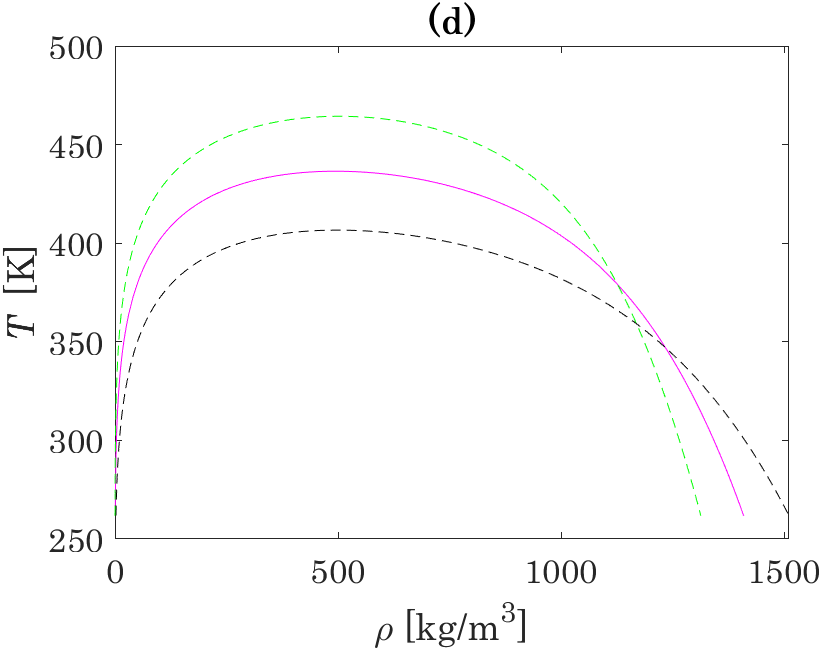


**Figure S1**. Results of sensitivity analysis performed for critical temperatures: (a) and (b) – for T_c_(NO_2_), (c) and (d) – for T_c_(N_2_O_4_).


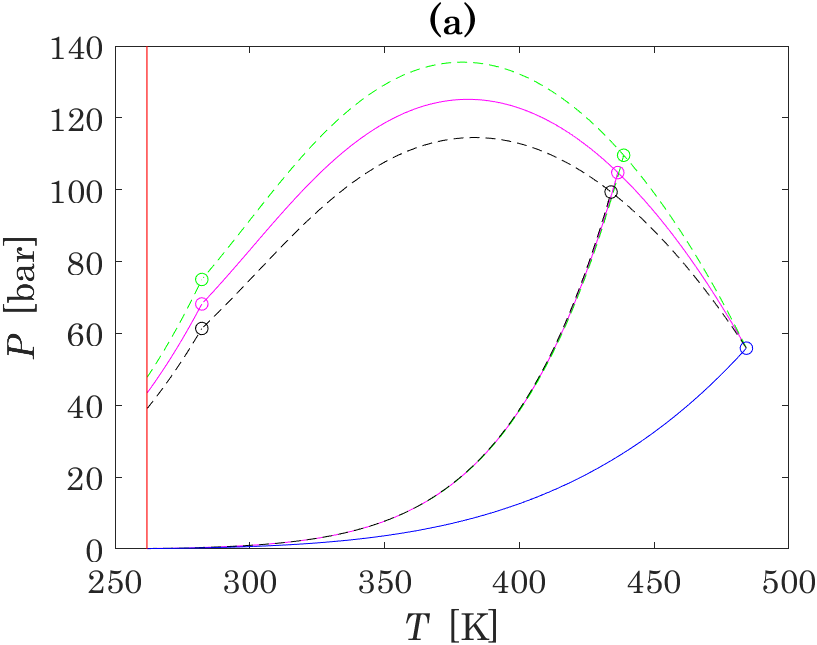

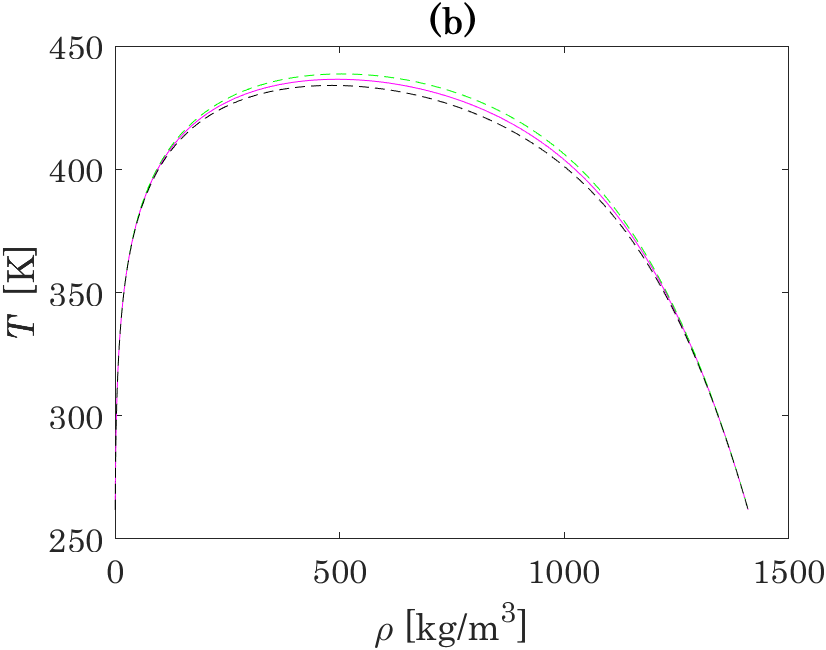


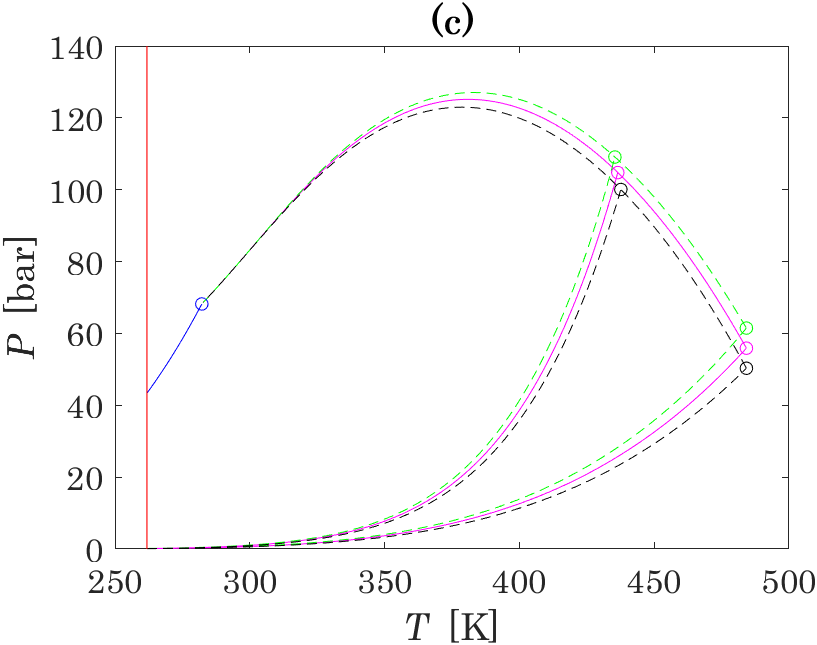

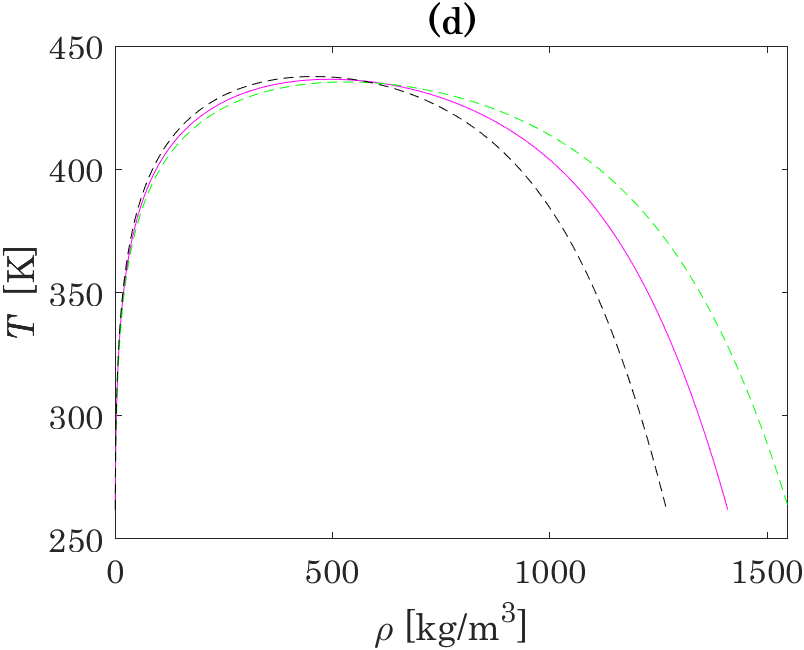


**Figure S2**. Results of sensitivity analysis performed for critical pressures: (a) and (b) – for P_c_(NO_2_), (c) and (d) – for P_c_(N_2_O_4_).


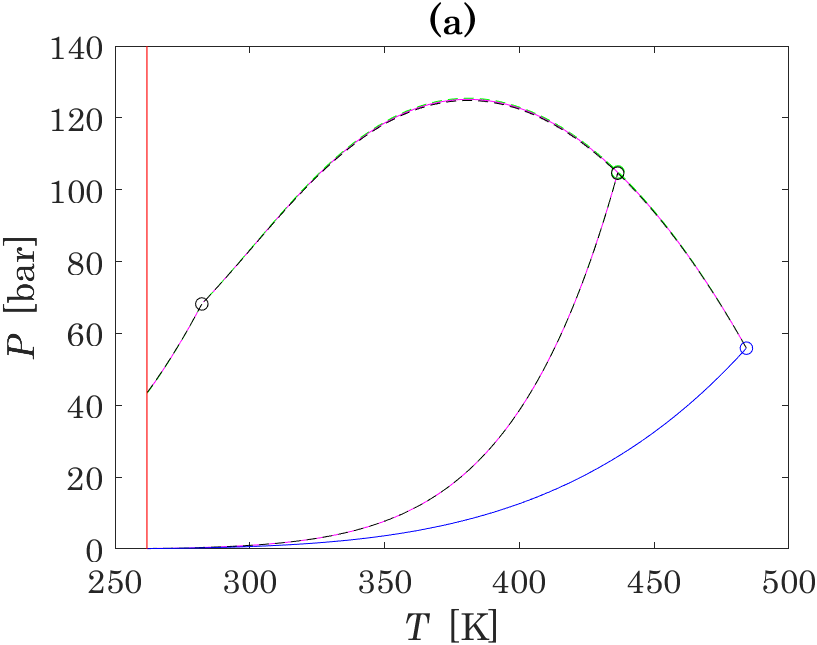

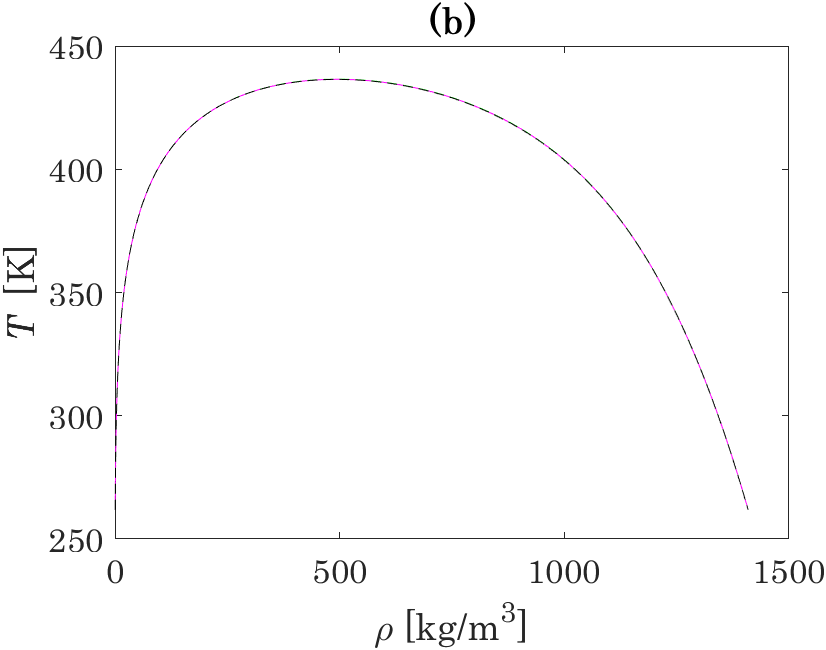


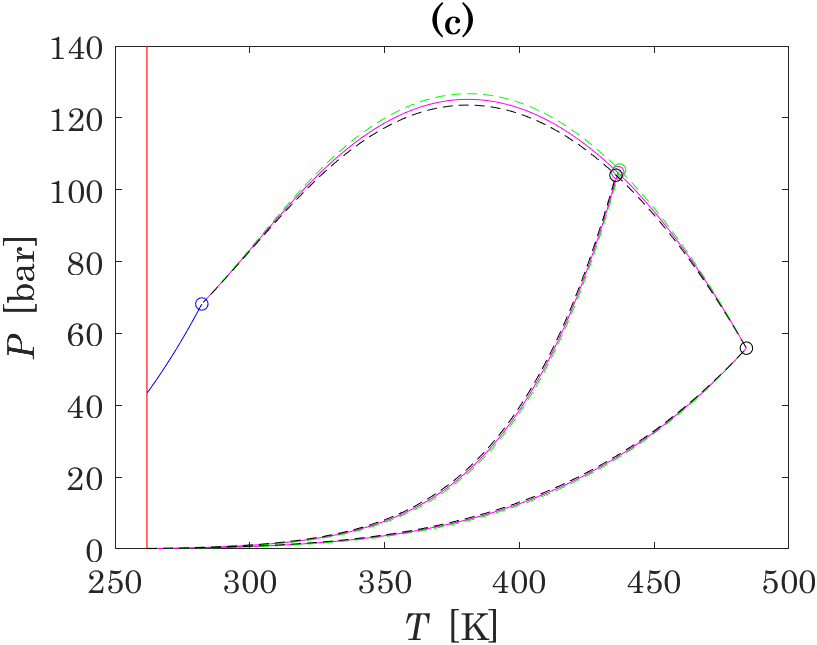

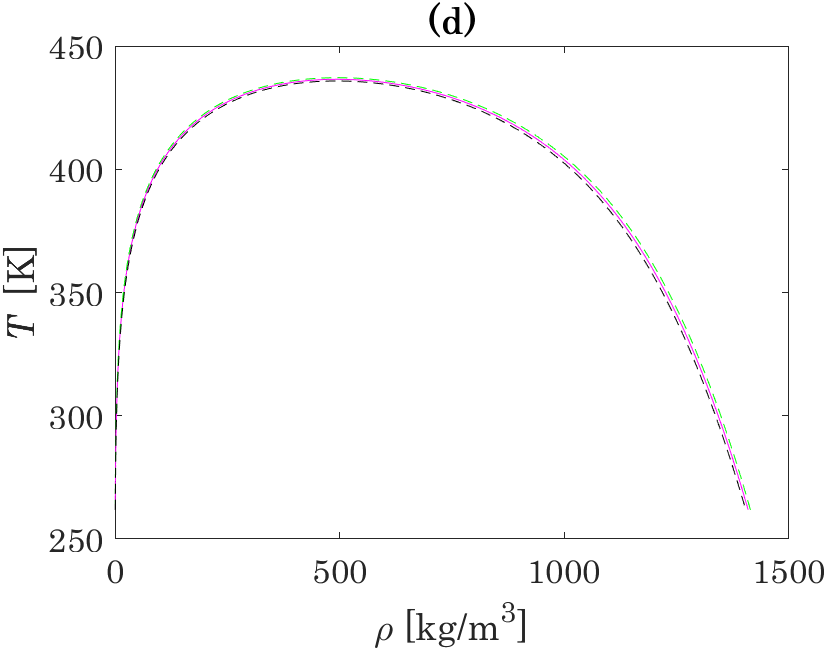


**Figure S3**. Results of sensitivity analysis performed for acentric factors: (a) and (b) – for ω(NO_2_), (c) and (d) – for ω(N_2_O_4_).


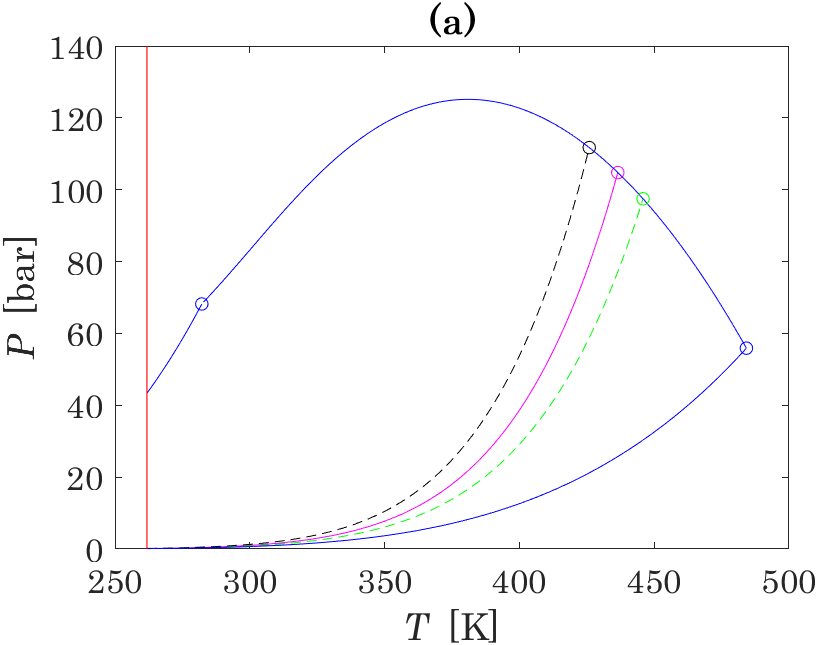

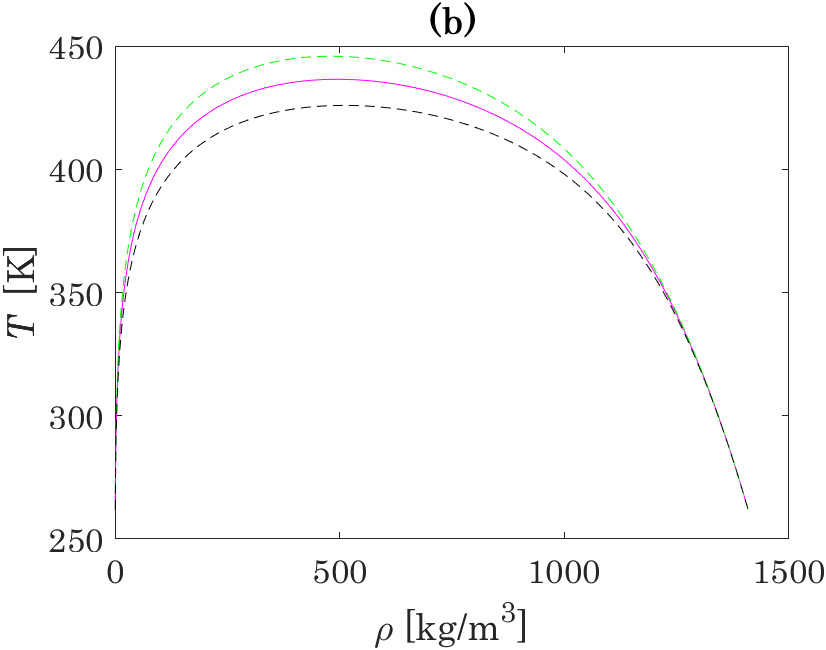


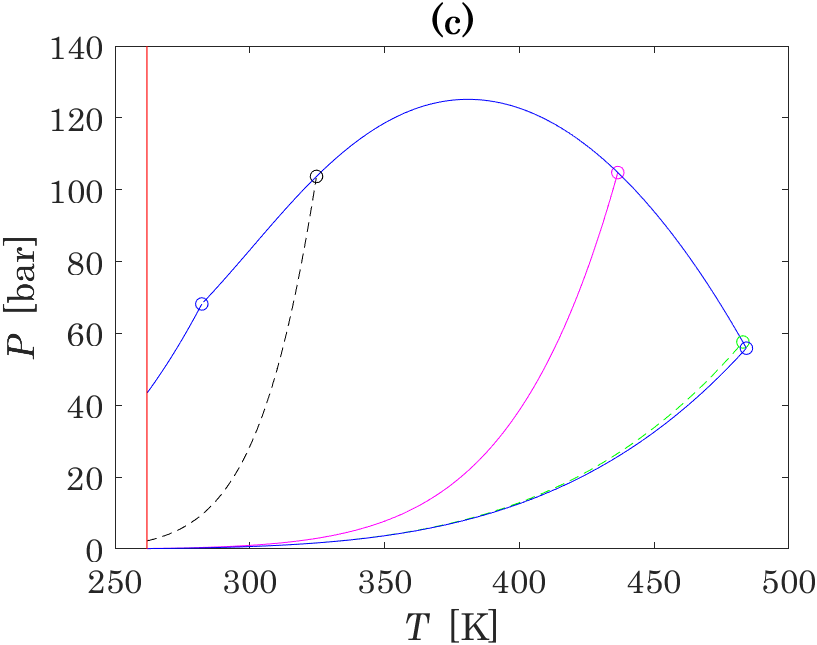

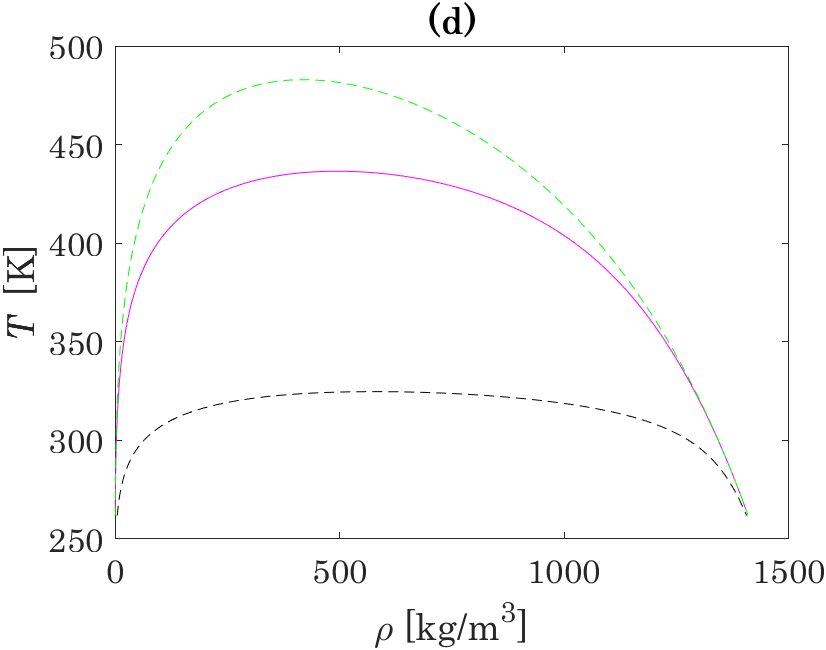


**Figure S4**. Results of sensitivity analysis performed for standard molar properties of the N_2_O_4_ ⇄ 2NO_2_ reaction: (a) and (b) – for $\Delta_{R}H_{298.15 K}^{\circ}$, (c) and (d) – for $\Delta_{R}S_{298.15 K}^{\circ}$.

**S4. Validity of the Clapeyron equation for reactive systems** **with congruent material transfer between phases**

Consider the change of the extensive Gibbs energy in the reactive system:

$$dG=-S\cdot dT+V\cdot dP+\Delta_{R}G\cdot d\xi$$

In the case of the phase equilibrium between liquid and vapor phases, we can write

$$0=dG^{vap}-dG^{liq}=-(S^{vap}-S^{liq})\cdot dT+(V^{vap}-V^{liq})\cdot dP+\Delta_{R}G^{vap}\cdot d\xi^{vap}-\Delta_{R}G^{liq}\cdot d\xi^{liq}$$

Since at chemical equilibrium the Gibbs energies of the reaction of vapor and of liquid are both equal to zero, we obtain:

$$0=\Delta V\cdot dP-\Delta S\cdot dT$$

$$\frac{dP}{dT}=\frac{\Delta S}{\Delta V}=\frac{\Delta H}{T\cdot\Delta V}$$

Thus, the Clapeyron equation is still valid for reactive systems. However, we can not rewrite eq. in terms of molar properties as in the case of inert fluids, since the composition of the system changes during the process of evaporation ($n^{vap}\neq n^{liq}$):

$$\Delta H=H^{vap}-H^{liq}=n^{vap}h^{vap}-n^{liq}h^{liq}$$

$$\Delta V=V^{vap}-V^{liq}=n^{vap}v^{vap}-n^{liq}v^{liq}$$

Since mass of the system does not change during the evaporation process ($m^{vap}=m^{liq}=m$), we can use the Clapeyron equation in terms of mass properties:

$$\Delta H=H^{vap}-H^{liq}=m^{vap}h_{mass}^{vap}-m^{liq}h_{mass}^{liq}=m(h_{mass}^{vap}-h_{mass}^{liq})=m\Delta h_{mass}$$

$$\Delta V=V^{vap}-V^{liq}=m^{vap}v_{mass}^{vap}-m^{liq}v_{mass}^{liq}=m(v_{mass}^{vap}-v_{mass}^{liq})=m\Delta v_{mass}$$

$$\frac{dP}{dT}=\frac{\Delta h_{mass}}{T\Delta v_{mass}}$$

**References**

[1] J. Chao, R.C. Wilhoit, B.J. Zwolinski, Gas Phase Chemical Equilibrium in Dinitrogen Trioxide and Dinitrogen Tetroxide, Thermochim. Acta 10 (1974) 359–371. https://doi.org/10.1016/0040-6031(74)87005-X.

[2] I.C. Hisatsune, Thermodynamic Properties of Some Oxides of Nitrogen, J. Phys. Chem. 65 (1961) 2249–2253. https://doi.org/10.1021/j100829a037.

[3] A.P. Altshuller, Thermodynamic Functions for Nitrogen Dioxide and Nitrous Acid, J. Phys. Chem. 61 (1957) 251–253. https://doi.org/10.1021/j150548a026.

[4] J.S. Gordon, Thermodynamic Functions of the Gaseous Dioxides of Carbon, Nitrogen, Sulfur, and Chlorine, and of Carbon Disulfide and Oxysulfide, J. Chem. Eng. Data 6 (1961) 390–394. https://doi.org/10.1021/je00103a022.

[5] S. Lasala, K. Samukov, H. Mert Polat, V. Lachet, O. Herbinet, R. Privat, J.-N. Jaubert, O. Moultos, K. de Ras, T. J. H. Vlugt, Application of Thermodynamics at Different Scales to Describe the Behaviour of Fast Reacting Binary Mixtures in Vapour-Liquid Equilibrium, Chem. Eng. J. 483 (2024) 148961. https://doi.org/10.1016/j.cej.2024.148961.

[6] L.V. Gurvič, I.V. Vejc, A.V. Medvedev, Termičeskie Konstanty Veŝestv [Thermal Constants of Substances]. Volume 1: Elements O, H(D,T), F, Cl, Br, I, He, Ne, Ar, Kr, Xe, Rn, S, N, P and Their Compounds. Book 2: Tables of Thermodynamic Properties, 3rd ed., Nauka, Moscow, 1978.

[7] NIST-JANAF Thermochemical Tables, (2013). https://janaf.nist.gov/ (accessed November 1, 2024).

[8] H. Blend, Study of Equilibria in Nitrogen Dioxide, J. Chem. Phys. 53 (1970) 4497–4499. https://doi.org/10.1063/1.1673979.

[9] L. Harris, K.L. Churney, Evaluation of the Equilibrium Constant for the N_2_O_4_ (g) = 2NO_2_ (g) Reaction at 298.16°K from Light-Transmission Measurements, J. Chem. Phys. 47 (1967) 1703–1709. https://doi.org/10.1063/1.1712153.

[10] L.V. Mišina, G.Z. Serebrânyj, S.A. Šalygin, Calculation of the Equilibrium Constant of the Reaction N_2_O_4_ ⇄ 2NO_2_ Based on the Molecular Constants of the Components, Vescì Akad. Navuk BSSR Seryâ Fiz.-Teh. Navuk (1967) 23–28.

[11] A.J. Vosper, Dissociation of Dinitrogen Tetroxide in the Gas Phase, J. Chem. Soc. Inorg. Phys. Theor. (1970) 625–627. https://doi.org/10.1039/j19700000625.

[12] E.D. Glendening, A.M. Halpern, *Ab Initio* Calculations of Nitrogen Oxide Reactions: Formation of N_2_O_2_, N_2_O_3_, N_2_O_4_, N_2_O_5_, and N_4_O_2_ from NO, NO_2_, NO_3_, and N_2_O, J. Chem. Phys. 127 (2007) 164307. https://doi.org/10.1063/1.2777145.

[13] K. Bennewitz, J.J. Windisch, Eine neue Methode zur Bestimmung der Dichte von aggressiven Flüssigkeiten unter hohem Druck, im besonderen der kritischen Dichte von Stickstofftetroxyd, Z. Für Phys. Chem. 166A (1933) 401–415. https://doi.org/10.1515/zpch-1933-16640.

[14] A.Ž. Greben’kov, V.P. Curbelev, S.V. Limarenko, Phase Equilibrium Parameters of Nitrogen Tetroxide and Their Asymptotic Behavior in the Critical Point Region. I. Vapour Pressure, Vescì Akad. Navuk BSSR Seryâ Fiz.-Energ. Navuk (1985) 56–60.

[15] A. Nadejdine, La Détermination de la Température Critique dans les Tubes Opaques, Bull. Acad. Imp. Sci. St-Petersbourg 30 (1886) 327–330. https://www.biodiversitylibrary.org/bibliography/49351.

[16] N.G. Polikhronidi, R.G. Batyrova, I.M. Abdulagatov, Isochoric Heat Capacity Measurements of Nitrogen Tetroxide System at Temperatures Between 410 and 484 K and Pressures Up to 35 MPa, Fluid Phase Equilibria 175 (2000) 153–174. https://doi.org/10.1016/S0378-3812(00)00457-X.

[17] F.E.C. Scheffer, J.P. Treub, Determinations of Vapour Tensions of Nitrogen Tetroxide, Proc. KNAW 14 (1911) 536–549. https://dwc.knaw.nl/toegangen/digital-library-knaw/?pagetype=publDetail&pId=PU00013111.

[18] F.E.C. Scheffer, J.P. Treub, Determinations of the Vapour Tension of Nitrogen Tetroxide, Proc. KNAW 15 (1912) 166–178. https://dwc.knaw.nl/toegangen/digital-library-knaw/?pagetype=publDetail&pId=PU00012961.

[19] F.E.C. Scheffer, J.P. Treub, Die Dampfdruckkurve des Stickstofftetroxyds, Z. Für Phys. Chem. 81U (1913) 308–332. https://doi.org/10.1515/zpch-1913-8116.

[20] H.H. Reamer, B.H. Sage, Volumetric Behavior of Nitrogen Dioxide in the Liquid Phase, Ind. Eng. Chem. 44 (1952) 185–187. https://doi.org/10.1021/ie50505a052.

[21] W.G. Schlinger, B.H. Sage, Volumetric Behavior of Nitrogen Dioxide, Ind. Eng. Chem. 42 (1950) 2158–2163. https://doi.org/10.1021/ie50490a042.

[22] O. Scheuer, Preliminary Report of the Examination of the Physical and Chemical Properties of Pure Gases and Binary Mixtures, Anz. Österr. Akad. Wiss. 48 (1911) 304–307. https://www.zobodat.at/publikation_volumes.php?id=32597.

[23] V.P. Curbelev, Determination of the Critical Temperature of Dissociating Nitrogen Tetroxide, Vescì Akad. Navuk BSSR Seryâ Fiz.-Energ. Navuk (1984) 58–60.

[24] V.P. Сurbelev, Temperature Dependency of Density on the Liquid-Gas Coexistence Curve for NO_2_-NO Solutions in the Extended Critical Region (Experimental Determination and Tables of Thermodynamic Properties), Abstract of a PhD thesis in Technical Sciences, Institute of Nuclear Energy of the Belarus Academy of Sciences, 1986.

[25] A.B. Veržinskaâ, V.P. Curbelev, P.M. Klepatskij, Impact of Nitrogen Monoxide on the Shape of the Coexistence Curve of N_2_O_4_ in the Vicinity of the Critical Point Liquid-Vapour, in: 5th -Union Sch. Appl. Math. Methods Descr. Stud. Phys.-Chem. Equilibria Ext. Abstr. Rep. Part II, Novosibirsk, 1985: pp. 120–123.

[26] V.B. Nesterenko, Ed. Teplofizičeskie Svojstva Četyrekhokisi Azota [Thermophysical Properties of Nitrogen Tetroxide], Nauka i Tehnika, Minsk, 1982.

[27] W.F. Giauque, J.D. Kemp, The Entropies of Nitrogen Tetroxide and Nitrogen Dioxide. The Heat Capacity from 15°K to the Boiling Point. The Heat of Vaporization and Vapor Pressure. The Equilibria N_2_O_4_=2NO_2_=2NO+O_2_, J. Chem. Phys. 6 (1938) 40–52. https://doi.org/10.1063/1.1750122.

[28] C.C. Addison, J.C. Sheldon, 373. Vapour Pressures of Mixtures of Dinitrogen Tetroxide with Donor and with Non-Donor Organic Solvents: Comparison with Some Nitrosyl Chloride Systems, J. Chem. Soc. (1957) 1937–1945. https://doi.org/10.1039/JR9570001937.

[29] G. Baume, M. Robert, Some Properties of Pure Dinitrogen Trioxide and of its Solution in Nitrogen Peroxide, Comptes Rendus Hebd. Séances Académie Sci. 169 (1919) 968–970. https://doi.org/10.1259/jrs.1912.0009.

[30] G. Baume, M. Robert, A Glass Manometer with Elastic Walls, Comptes Rendus Hebd. Séances Académie Sci. 168 (1919) 1199–1201. https://doi.org/10.1259/jrs.1912.0009.

[31] Ph.-A. Guye, G. Drouginine, Nouvelle Révision du Poids Atomique de l’Azote. Analyse Exacte du Peroxyde d’Azote, J. Chim. Phys. 8 (1910) 473–514. https://doi.org/10.1051/jcp/1910080473.

[32] A. Mittasch, E. Kuss, H. Schlueter, Dichten und Dampfdrucke von wäßrigen Ammoniaklösungen und von flüssigem Stickstofftetroxyd für das Temperatur-gebiet 0° bis 60°, Z. Für Anorg. Allg. Chem. 159 (1927) 1–36. https://doi.org/10.1002/zaac.19261590102.

[33] W. Ramsay, S. Young, On Evaporation and Dissociation. Part I, Philos. Trans. R. Soc. Lond. 177 (1886) 71–122. https://doi.org/10.1098/rstl.1886.0003.

[34] F.T. Selleck, H.H. Reamer, B.H. Sage, Volumetric and Phase Behavior of Mixtures of Nitric Oxide and Nitrogen Dioxide, Ind. Eng. Chem. 45 (1953) 814–819. https://doi.org/10.1021/ie50520a046.

[35] E.M. Stoddart, The Effect of Drying on the Vapour Pressure of Dinitrogen Tetroxide and the Vapour Density of Dinitrogen Trioxide, J. Chem. Soc. Resumed (1945) 448. https://doi.org/10.1039/jr9450000448.

[36] T.E. Thorpe, On the Relation Between the Molecular Weights of Substances and Their Specific Gravities When in the Liquid State, J. Chem. Soc. Trans. 37 (1880) 141–225. https://doi.org/10.1039/CT8803700141.

[37] V.A. Cymarnyj, Density of Liquid Nitrogen Tetroxide at Temperatures 300-500 K and Pressures Up to 600 Bar, N. 2165-70, Deposited in VINITI, Odessa, 1970.

[38] P. Pascal, M. Garnier, Relations Between Nitrogen Peroxide and Nitric Acid, Bull. Société Chim. Fr. 25 (1919) 309–321.

[39] A.Ž. Greben’kov, V.P. Curbelev, A.B. Veržinskaâ, V.B. Nesterenko, Study of the Thermodynamic Properties of Nitrogen Dioxide in the Wide Range of the Critical Point, Teplofiz. Svojstva Veŝestv Mater. 27 (1989) 41–53.

[40] H.I. Amirhanov, N.G. Polihronidi, B.G. Alibekov, R.G. Batyrova, Isochoric Heat Capacity of Nitrogen Tetroxide and the Effect of Dissociation on the Form of *C*_v_(*T*), Vescì Akad. Navuk BSSR Seryâ Fiz.-Energ. Navuk (1981) 113–118.

[41] A.B. Veržinskaâ, V.P. Curbelev, Experimental Study of *T*_s_-*ρ*_s_ Parameters of Dissociated Heat-Transport Medium on the Basis of N_2_O_4_ by the Method of Quasi-Static Thermogram’s in the Range of Temperature 373 K to *T*_c_, Vescì Akad. Navuk BSSR Seryâ Fiz.-Energ. Navuk (1982) 43–46.

[42] P. Gray, P. Rathbone, Dissociation of Liquid Dinitrogen Tetroxide; Henry’s Law Coefficients, Heats and Entropies of Solution, and the Thermodynamics of Homolytic Dissociation in the Pure Liquid, J. Chem. Soc. (1958) 3550. https://doi.org/10.1039/jr9580003550.

[43] D.W. James, R.C. Marshall, Electron Spin Resonance Study of the Dinitrogen Tetroxide-Nitrogen Dioxide System, J. Phys. Chem. 72 (1968) 2963–2966. https://doi.org/10.1021/j100854a048.

[44] A.J. Vosper, The Dissociation of Dinitrogen Tetroxide in the Liquid Phase, J. Chem. Soc. Inorg. Phys. Theor. (1970) 2191–2193. https://doi.org/10.1039/j19700002191.

[45] C.C. Addison, B.C. Smith, Volume Changes on Mixing Organic Liquids with Dinitrogen Tetroxide: Comparison with Sulphur Dioxide Systems, J. Chem. Soc. Resumed (1958) 3664. https://doi.org/10.1039/jr9580003664.

[46] W. Ramsay, J. Shields, LXXXI.—The Molecular Complexity of Liquids, J. Chem. Soc. Trans. 63 (1893) 1089–1109. https://doi.org/10.1039/CT8936301089.

[47] W.R. Bousfield, III.—Mixtures of Nitrogen Peroxide and Nitric Acid, J. Chem. Soc. Trans. 115 (1919) 45–55. https://doi.org/10.1039/CT9191500045.

[48] L.S. Vitûk, Èksperimental’naâ Metodika i Rezul’taty Issledovaniâ Termodinamičeskih Svojstv Agressivnyh i Vâzkih Veŝestv Kak Rabočih Tel Ènergetičeskih Ustanovok [Experimental Methodology and Results of the Determination of Thermodynamic Properties of Aggressive and Viscous Compounds as Working Fluids of Power Plants, Abstract of a PhD thesis in Technical Sciences, Odessa Technological Institute of Refrigeration Industry, 1982.

[49] L.S. Vitûk, E.A. Golovskij, Experimental Investigation of Density and Isobaric Heat Capacity of Nitrogen Tetroxide and Solutions Based on It, Teplofiz. Svojstva Veŝestv Mater. (1988) 40–48.

[50] L.S. Vitûk, E.A. Golovskij, A.G. Tabačnikov, Experimental Study of Thermodynamic Properties of Nitrogen Tetroxide at 230-330 K, Deposited in ONIITEKHim, SPSTL 39 Khp-D80, (1980).

[51] Û.A. Sarumov, Issledovanie Èntalʹpii Azotnogo Tetraksida v Širokoj Oblasti Parametrov Sostoâniâ [Investigation of Enthalpy of Nitrogen Tetroxide in a Wide Range of State Parameters], Abstract of a PhD thesis in Technical Sciences, Moscow Energy Institute of the Order of Lenin, 1969.

[52] А.Е. Šejndlin, N.I. Gorbunova, Û.A. Sarumov, Experimental Investigation of Enthalpy of Chemically Reacting System N_2_O_4_ ⇆ 2NO_2_ ⇆ 2NO + O_2_, Dokl. Akad. Nauk SSSR 186 (1969) 817–819. https://www.mathnet.ru/links/b618abf372d953d4811aeb9a595d0417/dan34675.pdf.

[53] А.Е. Šejndlin, N.I. Gorbunova, Û.A. Sarumov, Corrections to the Article “Experimental Investigation of Enthalpy of Chemically Reacting System N_2_O_4_ ⇆ 2NO_2_ ⇆ 2NO + O_2_” (Doklady Akademii Nauk SSSR, 186(4), 1969), Dokl. Akad. Nauk SSSR 188 (1969) 8. https://www.mathnet.ru/links/ee8d87bbf438cbdfad446aa19d314cd0/dan34919.pdf.

[54] А.Е. Šejndlin, N.I. Gorbunova, Û.A. Sarumov, Enthalpy of Nitrogen Tetroxide in the near-Critical Region of the Parameters of State, Teplofiz. Vysok. Temp. 11 (1973) 1192–1197. https://www.mathnet.ru/links/ad86d58a9bd82c9671a4d585c4eb8611/tvt9976.pdf.

[55] А.Е. Šejndlin, N.I. Gorbunova, V.M. Simonov, Experimental Enthalpy Data of Nitrogen Tetroxide, Teplofiz. Vysok. Temp. 12 (1974) 666–669. https://www.mathnet.ru/links/392bc0b823e30348eaf9121a2d4d64b6/tvt9397.pdf.

[56] А.Е. Šejndlin, N.I. Gorbunova, V.M. Simonov, Enthalpy of Dissociating Nitrogen Tetroxide at Pressures Up to 30 MPa and Temperatures Up to 782 K, Teplofiz. Vysok. Temp. 15 (1977) 767–771. https://www.mathnet.ru/links/125b26aa45934caf22230266a7698229/tvt7139.pdf.

[57] V.M. Simonov, Èksperimentalʹnoe Issledovanie Kaloričeskih Svojstv Četyrehokisi Azota i Difenilʹnoj Smesi [Experimental Investigation of Caloric Properties of Nitrogen Tetroxide and Diphenyl Mixture], Abstract of a PhD thesis in Technical Sciences, Institute of High Temperatures, Academy of Sciences of the USSR, 1977.

1. Any mention of commercial products within this article is for information only; it does not imply recommendation or endorsement by NIST. These opinions, recommendations, findings, and conclusions do not necessarily reflect the views or policies of NIST or the United States Government. [↑](#footnote-ref-1)
